# Temporal trends of particulate matter pollution and its health burden, 1990–2021, with projections to 2036: a systematic analysis for the global burden of disease study 2021

**DOI:** 10.3389/fpubh.2025.1579716

**Published:** 2025-04-16

**Authors:** Tao Fang, Yanbo Di, Yang Xu, Na Shen, Haojun Fan, Shike Hou, Xiaoxue Li

**Affiliations:** ^1^Institute of Disaster and Emergency Medicine, Tianjin University, Tianjin, China; ^2^The Fourth Central Hospital, Medical School of Tianjin University, Tianjin University, Tianjin, China; ^3^Central Laboratory, Tianjin 4th Center Hospital, Tianjin, China; ^4^Department of Medicine, Tianjin Medical College, Tianjin, China; ^5^Disaster Medicine Research Center, Medical Innovation Research Division of the Chinese PLA General Hospital, Beijing, China; ^6^2021RU006 Research Unit of Disaster Medicine, Chinese Academy of Medical Sciences, Beijing, China; ^7^Beijing Key Laboratory of Disaster Medicine, The Chinese PLA General Hospital, Beijing, China

**Keywords:** burden of disease, particulate matter pollution, PM2.5, temporal trends, Level-3 diseases, projection

## Abstract

**Background:**

Particulate matter pollution (PM2.5) is a leading global health risk factor. We analyzed the spatiotemporal trends of diseases attributable to PM2.5 at global, regional, and national levels from 1990 to 2021.

**Methods:**

Using data from the Global Burden of Disease (GBD) 2021 study, we assessed global, regional, and national deaths and disability-adjusted life years (DALYs) due to PM2.5, along with age-standardized mortality rates (ASMR) and age-standardized DALY rates (ASDR), categorized by age, sex, year, location, and disease type. We used average annual percentage change (AAPC) to illustrate trends from 1990 to 2021. Spearman correlation analysis was conducted to examine the relationship between the socio-demographic index (SDI) and age-standardized rates (ASRs) across 204 countries. Bayesian age-period-cohort (BAPC) analysis was used to project trends for 2022–2036.

**Results:**

In 2021, PM2.5 exposure contributed to 7.83 million deaths and 231.51 million DALYs globally. The age-standardized rates decreased to 95.69 per 100,000 for deaths (AAPC = −2.12) and 2984.47 per 100,000 for DALYs (AAPC = −2.22), compared to 1990. Disease burdens related to PM2.5, as reflected by ASMR and ASDR, declined across SDI quintiles and GBD super regions from 1990 to 2021. The low SDI quintile had the highest disease burden (ASMR: 211.39, ASDR: 6,114.26). Correlation analysis revealed a significant negative relationship between ASRs and SDI. South Asia and sub-Saharan Africa experienced the highest disease burdens. Males had higher disease burdens than females globally and in all regions. The burden was particularly severe for children under five and older adults. Ischemic heart disease and stroke were the leading causes of PM2.5-related deaths and DALYs. Diabetes mellitus saw an increase in both deaths and DALYs. The BAPC model predicts continued declines in PM2.5-related ASDR and ASMR over the next 15 years.

**Conclusion:**

With population growth and an aging demographic, the public health burden associated with PM2.5 exposure remains a major concern. It is imperative to develop targeted and proactive strategies that account for the unique circumstances and challenges of different regions.

## 1 Introduction

The advancement of modern society has brought about major concerns over particulate matter pollution (specifically PM2.5), which poses a significant global environmental health risk and contributes substantially to mortality and disease burden. PM2.5, defined as fine particles with a diameter of less than 2.5 μm, can penetrate the alveoli and enter the bloodstream, thereby affecting vital organs and precipitating various health issues (1). Although the underlying biological mechanisms are not fully understood, a growing body of research suggests that both long-term and short-term exposure to PM2.5 is associated with a variety of diseases, including cardiovascular diseases (e.g., ischemic heart disease and stroke), preterm birth, respiratory disorders, and lung cancer (2–5). According to the latest risk factor analysis of Global Burden of Disease (GBD) 2021, particulate matter pollution, including both ambient and household air pollution, is the leading level 3 risk factor globally, contributing 8.0% (ranging from 6.7–9.4%) of total disability-adjusted life years (DALYs) (6). The impact of PM2.5 pollution varies across different regions, countries, and socioeconomic groups, with low- and middle-income countries (LMICs) bearing a disproportionately higher burden. This disparity highlights the urgent need for policy and health interventions (7).

PM2.5 is known for its complex composition and diverse sources. Since air pollution and atmospheric chemistry transcend geographical boundaries, mitigation strategies must consider cross-border impacts. To develop effective policies and interventions, it is essential to incorporate research on the disease burden associated with PM2.5 exposure from sub-national to global levels (8). Bu et al. examined the differences in global disease burdens between ambient and household PM2.5 using GBD 2017 data. In another study, Sang et al. analyzed the global and regional disease burdens linked to ambient PM2.5 using GBD 2019 data (9, 10). However, there is a lack of comprehensive time trend analyses of PM2.5 across various regions, sexes, and disease types based on GBD 2021 data. Such analyses are crucial for improving our understanding of the health impacts of air pollution and provide valuable insights to guide the development of effective environmental policies and governance strategies.

The most recent GBD risk factor analysis incorporated the burden of proof function (BPRF) methods to improve standard estimates of risk-outcome relationships, update the GBD mediation matrix, and optimize the computational process by reducing the number of calculations (6). In this study, we utilized the latest GBD 2021 dataset to conduct a comprehensive analysis of the disease burden attributable to PM2.5 pollution, disaggregated by location, sex, and age. Additionally, this study explores the changing patterns of PM2.5-attributed disease burden from 1990 to 2021, offering insights into the temporal shifts in mortality and DALY rates, with trend projections up to 2036.

## 2 Methods

### 2.1 Study data

The disease burden data attributable to PM2.5 from 1990 to 2021 were obtained from the Global Health Data Exchange (GHDX) GBD Results Tool,^1^ which systematically organizes epidemiological estimates for 88 risk factors and their associated health outcomes across 204 countries and territories. The GBD 2021study assessed the burden of risk factors using DALYs and deaths (6). DALYs represent the total years of life lost due to premature mortality and the years lived with disability (11). The burden of disease associated with PM2.5 was quantified using the number and age-standardized rates (ASRs) of death and DALYs. Estimates for all metrics were computed by taking the mean estimates across 500 draws, and 95% uncertainty intervals (UIs) are represented as the 2.5^th^ and 97.5^th^ percentiles of that distribution. The age range in this study included individuals from <5 years to ≥95 years, grouped into 20 categories, each spanning 5 years. Particulate matter pollution is classified as a level 3 risk factor in the GBD 2021 study while encompassing two level 4 risk factors: ambient fine particulate pollution and household air pollution from solid fuels. The GBD 2021 classifies particulate matter pollution as a level 3 risk factor, with a focus on PM2.5 as the main indicator of health risk. It standardizes data from other particulate matter pollutants, such as PM10, to PM2.5-equivalent measurements to ensure comparable risk assessments. Detailed information on the estimation workflow and statistical methods for PM2.5-attributed health burden in the GBD 2021 study has been previously published (6) and can be accessed at the following websites: https://ghdx.healthdata.org/gbd-2021/code/risk-1 and https://ghdx.healthdata.org/gbd-2021/code/risk-2.

Diseases and injuries in the GBD 2021 study are classified into four mutually exclusive levels. Level-1diseases includes the three most common causes of death and disability, while Level-4 diseases contains the most specific classifications. This study identified 13 Level-3 diseases to evaluate the disease burden attributable to PM2.5. These diseases include chronic obstructive pulmonary disease (COPD), diabetes mellitus (DM), diarrheal diseases, encephalitis, ischemic heart disease (IHD), lower respiratory infections (LRI), meningitis, neonatal disorders, otitis media, stroke, tracheal, bronchus and lung cancer, sudden infant death syndrome and upper respiratory infections. Blindness and visual impairment, also classified as Level-3 diseases, were excluded from the analysis due to the lack of mortality data.

GBD regions consist of countries and territories that are geographically close and epidemiologically similar. These regions are further grouped into super regions based on patterns of cause-specific mortality. The seven super regions are: High-income; Southeast Asia, East Asia, and Oceania; Central Europe, Eastern Europe, and Central Asia; Latin America and the Caribbean; North Africa and Middle East; South Asia; Sub-Saharan Africa. The socio-demographic index (SDI) is a composite measure of a country’s development status, ranging from 0 to 1. It is based on criteria such as fertility rates among women under 25, mean educational attainment for individuals aged 15 and older, and lag-distributed income per capita (12). All 204 countries and territories are assigned an SDI value and grouped into quintiles that reflect low to high development levels. Data related to epidemiological and geographical conditions from 204 countries and territories, five SDIs, and seven GBD super regions were used to estimate the disease burden. We have organized the above information in Supplementary Table S1 based on the latest GBD data (6).

The institutional ethics committee granted an exemption for this study, as the data from GBD 2021 are publicly available. This study followed the guidelines for accurate and transparent health assessment reporting.

### 2.2 Statistical analyses

The average annual percentage change (AAPC) and its corresponding 95% confidence interval (CI) were calculated using the Joinpoint Regression Program (National Cancer Institute, Rockville, MD, USA) to quantify the secular trends of age-standardized mortality rates (ASMR) and age-standardized DALY rates (ASDR) from 1990 to 2021. The logarithmic age-standardized indicators were fitted to a regression model, $\ln\left(y\right)=a+\beta x+\varepsilon \text{,}$ where x represents the calendar year and y represents the respective age-standardized indicators. AAPC and its 95% CI was calculated based on the model, 100 × [exp (β) – 1] (13). An increasing trend in the age-standardized indicator was identified if the 95% CI of the corresponding AAPC estimate was greater than 0, while a decreasing trend was identified if the 95% CI was less than 0, and a stable trend if the 95% CI included 0. We conducted the Spearman correlation analysis between the SDI and the ASRs to examine the association between socio-demographic development and the PM2.5-attributable disease burden across 204 countries and territories. Bayesian age-period-cohort (BAPC) analyses were conducted using R (version 4.4.2) with the aid of the BAPC and INLA software packages (14). This allowed us to project ASRs by location from 2022 to 2036. Population forecast data can be accessed at the following website: https://ghdx.healthdata.org/record/ihme-data/global-population-forecasts-2017-2100. As the forecast data did not include SDI, this study predicted the ASRs across seven GBD super regions. A *p*-value <0.05 was considered statistically significant.

## 3 Results

### 3.1 The global health burden attributable to PM2.5 in 2021

In 2021, the global number of deaths attributable to PM2.5 was 7,833,221 (95% UI: 6,479,474–9,263,395), with an ASMR of 96.69 (79.91–114.4) per 100,000 people. There were 231,511,233 (194,538,892–270,855,451) DALYs attributable to PM2.5, with an ASDR of 2,984.47 (2,489.63–3,487.35) per 100,000 people. Males had a higher burden of deaths and DALYs compared to females, both in total numbers and ASRs (Table 1).

**Table 1:** Global deaths and DALYs attributable to PM2.5 in 1990 and 2021,and the temporal tread from 1990 to 2021.

Measure	Gender	1990		2021		1990–2021
		All-ages cases	ASR per 100,000	All-ages cases	ASR per 100,000	AAPC in ASR
		*n* (95%UI)	*n* (95%UI)	*n* (95%UI)	*n* (95%UI)	(95%CI)
Deaths	Both	7,250,210 (6,032,279–8,453,898)	182.73 (153.27–211.42)	7,833,221 (6,479,474–9,263,395)	96.69 (79.91–114.4)	−2.12^*^ (−2.40–−1.83)
	Female	3,382,535 (2,769,988–3,976,064)	155.45 (128.50–181.36)	3,478,614 (2,842,904–4,127,414)	78.35(64.30–92.70)	−2.24^*^ (−2.47–−2.02)
	Male	3,867,675 (3,238,507–4,480,011)	218.25 (183.44–252.10)	4,354,607 (3,629,597–5,195,554)	119.23 (99.30–142.09)	−1.97^*^ (−2.16–−1.78)
DALYs	Both	289,354,269 (222,295,153–350,589,635)	5,865.15 (4,690.12–6,998.3)	231,511,233 (194,538,892–270,855,451)	2,984.47(2,489.63–3,487.35)	−2.22^*^ (−2.39–−2.05)
	Fe	130,510,934 (96,977,293–160,665,648)	5,130.84 (3,925.85–6,220.82)	99,047,039 (82,679,827–117,756,359)	2,480.23 (2,059.40–2,951.19)	−2.36^*^ (−2.57–−2.16)
	Male	158,843,335 (124,606,242–190,518,631)	6,706.07 (5,501.04–7,859.56)	132,464,194 (110,995,51–154,576,904)	3,535.8 (2,953.30–4,130.72)	−2.12^*^ (−2.37–−1.87)

ASR, age-standardized rate; UI, uncertainty interval; AAPC, average annual percentage change; CI, confidential interval; DALYs, disability-adjusted life years; ^*^, *p* < 0.05.

**Figure 1:**
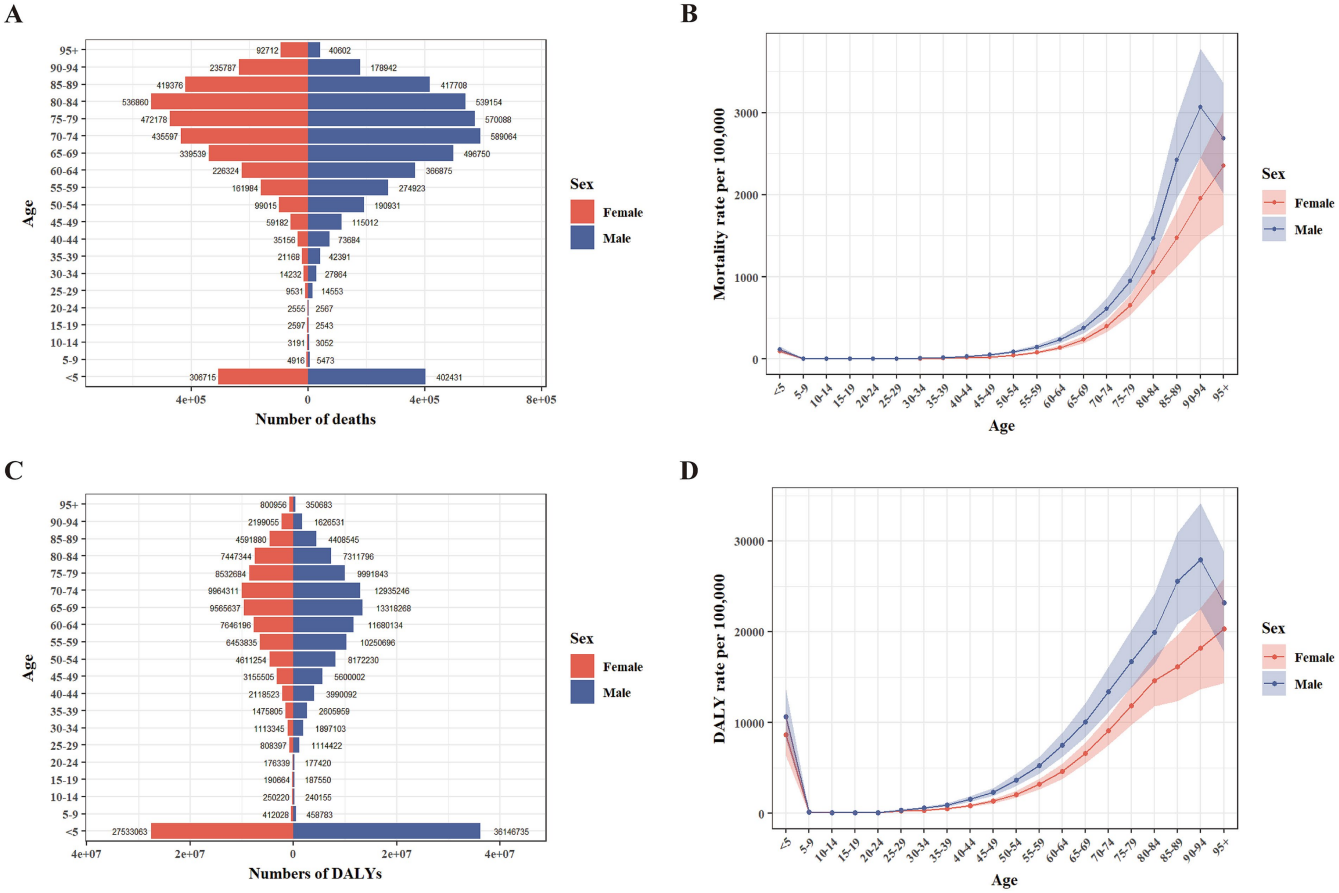
Age-specific numbers and rates of deaths and DALYs attributable to PM2.5 in 2021 by sex. **(A)** Number of deaths. **(B)** Rate of deaths. **(C)** Number of DALYs. **(D)** Rate of DALYs. DALYs, disability-adjusted life years.

Peaks were observed in age-specific death numbers: One in children <5 years for both sexes, one in the 70–74 age group for males, and one in the 80–84 age group for females (Figure 1A). DALYs pattern showed a similar trend. Both sexes had peaks <5 years, while the other peak occurred in 65–69 age group for males and 70–74 age group for females (Figure 1C). In the age groups of 10–14, 15–19, and > 85, the number of female deaths surpassed that of their male counterparts. Conversely, in all other age groups, mortality among males were higher (Figure 1A). In a similar manner, females exhibited a greater DALYs compared to males within the age groups of 10–14, 15–19, and >80, whereas males had a higher DALY in all other age categories (Figure 1C). The age-specific mortality and DALY rates were higher in females for the 10–14, 15–19, and 20–24 age groups but higher in males for all other age groups (Figures 1B,D). After the age of 20, the rates for both sexes increased. However, the rates for males started to decrease in the 90–94 age group, whereas the rates for females continued to rise (Figures 1B,D).

### 3.2 The health burdens of PM2.5 across regions and countries in 2021

For the SDI quintiles, middle SDI quintile had the largest number of PM2.5-attributable deaths (2,591,253 [95% UI: 2,065,517–3,185,581]), and low-middle SDI quintile had the largest number of DALYs (77,433,512 [64,788,178–90,113,245]). The highest ASMR (211.39 [174.87–245.86]) and ASDR (6,114.26 [5,006.33–7,220.39]) occurred in the low SDI quintile. Across the seven GBD super regions, Southeast Asia, East Asia, and Oceania had the most PM2.5-attributable deaths (3,015,190 [2,399,021–3,778,219]). Notably, South Asia had the most DALYs (80,560,949 [68,745,930–92,313,537]), the highest ASMR (193.96 [163.84–221.42]) and the highest ASDR (5,480.42 [4,694.75–6,270.19]). The ASMR (170.27 [136.87–204.29]) and ASDR (5,182.71 [4,128.54–6,309.84]) in sub-Saharan Africa ranked second among the seven super regions, indicating that this area also bears a heavy burden of disease. In all SDI quintiles and seven GBD super regions, the numbers and ASRs of death and DALYs were higher in males than in females (Supplementary Tables S2, S3).

**Figure 2:**
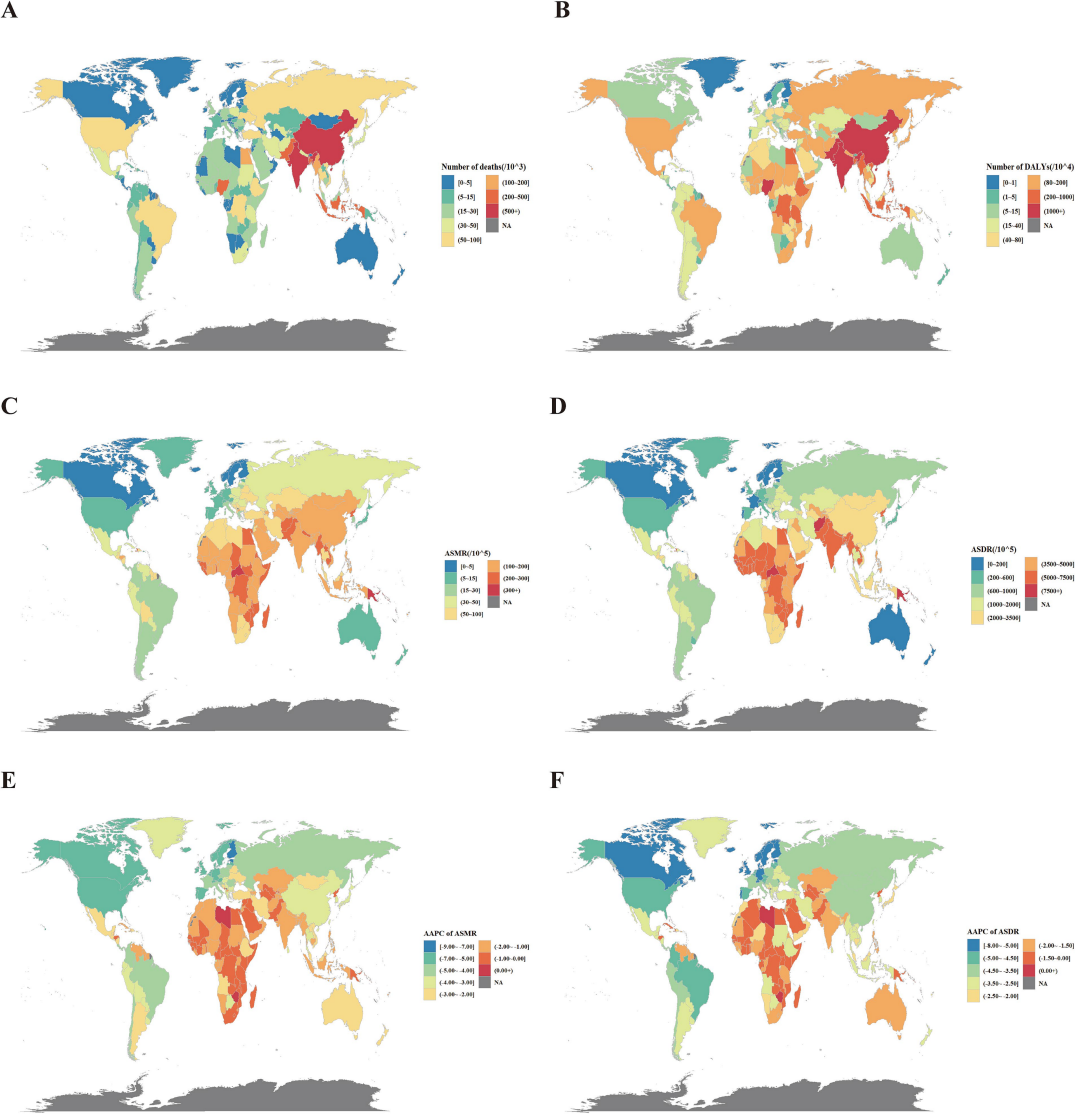
Global disease burden attributable to PM2.5 for both sexes. **(A)** Number of deaths in 2021. **(B)** Number of DALYs in 2021. **(C)** ASMR in 2021. **(D)** ASDR in 2021. **(E)** AAPC of ASMR from 1990 to 2021. **(F)** AAPC of ASDR in 2021. DALYs, disability-adjusted life years; ASMR, age-standardized mortality rates; ASDR, age-standardized DALY rates; AAPC, average annual percentage change.

For countries and territories, China (2,273,438 [1,771,097–2,892,737]), India (1,995,558 [1,679,989–2,304,834]), and Pakistan (250,887 [210,161–301,849]) ranked the highest globally in death numbers attributable to PM2.5. In terms of DALYs, India (60,941,687 [51,445,179–70,020,271]) surpassed China (46,676,903 [36,578,694–59,744,053]), making it the country with the highest disease burden, and Nigeria (12,797,073 [9,361,249–16,639,381]) was in third place. The highest ASMRs were observed in the Solomon Islands (360.85 [285.77–459.42]), Vanuatu (321.20 [262.18–379.08]), and the Central African Republic (317.43 [233.68–405.23]). ASDRs were the highest in the Central African Republic (9,427.06 [6,741.52–12,143.36]), Solomon Islands (8,813.39 [6,860.11–11,307.05]), and Guinea-Bissau (8,039.92 [6,189.7–9,800.33]) (Supplementary Table S4; Figures 2A–D).

### 3.3 The health burden of PM2.5 across Level-3 diseases in 2021

Among the 13 Level-3 diseases, IHD (2,492,810 [95% UI: 1,866,678–3,103,230]), stroke (1,989,686 [1,530,479–2,493,238]), COPD (1,535,298 [1,214,704–1,918,256]), LRI (651,238 [121,605–1,076,503]), and neonatal disease (496,966 [419,486–580,880]) accounted for the highest number of deaths. IHD (29.88 [22.33–37.22]), stroke (23.74 [18.26–29.80]), COPD (18.51 [14.64–23.13]), LRI (8.68 [1.71–14.39]), and neonatal disease (8.03 [6.78–9.39]) also ranked as the top 5 diseases in terms of ASMR (Table 2). The highest DALYs were found in IHD (54,675,670 [41,652,489–67,418,886]), stroke (44,962,167 [35,020,339–55,467,024]), neonatal disease (44,737,311 [37,766,690–52,293,054]), COPD (33,238,712 [26,680,066–41,336,741]), and LRI (29,098,331 [6,988,265–48,127,683]). On the other hand, ASDR was found to be the highest in neonatal diseases (723.06 [610.39–845.18]), followed by IHD (638.48 [486.47–787.82]), stroke (523.30 [407.96–645.58]), LRI (420.09 [106.35–693.12]), and COPD (389.50 [312.59–484.62]) (Supplementary Table S5).

**Table 2:** Global death of diseases attributable to PM2.5 in 1990 and 2021, and the temporal trend from 1990 to 2021.

Disease type	1990		2021		1990–2021
	Deaths *n* (95% UI)	ASMR per 100,000 *n* (95% UI)	Deaths *n* (95% UI)	ASMR per 100,000 *n* (95% UI)	AAPC in ASMR (95% CI)
Ischemic heart disease	1,570,387 (1,199,838–1,966,478)	45.07 (33.95–56.58)	2,492,810 (1,866,678–3,103,230)	29.88 (22.33–37.22)	−1.41^*^ (−1.60 –−1.22)
Stroke	1,755,017 (1,434,139–2,094,574)	48.86 (39.69–58.76)	1,989,686 (1,530,479–2,493,238)	23.74 (18.26–29.80)	−2.37^*^ (−2.63–−2.11)
Chronic obstructive pulmonary disease	1,490,216 (1,238,034–1,698,894)	42.53 (35.33–48.43)	1,535,298 (1,214,704–1,918,256)	18.51 (14.64–23.13)	−2.70^*^ (−3.00–−2.41)
Lower respiratory infections	1,211,024 (270,570–1,918,459)	23.35 (4.95–37.23)	651,238 (121,605–1,076,503)	8.68 (1.71–14.39)	−3.18^*^ (−3.41–−2.94)
Upper respiratory infections	247 (57–515)	<0.01 (<0.01–0.01)	117 (18–299)	<0.01 (<0.01–<0.01)	−2.34^*^ (−2.40–−2.27)
Otitis media	13 (4–31)	<0.01 (<0.01–<0.01)	2 (1–5)	<0.01 (<0.01–<0.01)	−6.14^*^(−6.33–−5.96)
Neonatal disorders	796,666 (729,078–862,650)	12.45 (11.39–13.48)	496,966 (419,486–580,880)	8.03 (6.78–9.39)	−1.42^*^ (−1.53–−1.31)
Diabetes mellitus	117,055 (69,992–165,962)	3.19 (1.91–4.55)	281,909 (165,678–395,528)	3.32 (1.95–4.66)	0.14^*^ (0.04–0.25)
Tracheal, bronchus, and lung cancer	253,291 (163,149–345,123)	6.39 (4.12–.71)	374,213 (236,358–520,255)	4.34 (2.74–6.04)	−1.28^*^ (−1.54–−1.02)
Diarrheal diseases	45,851 (32,228–56,815)	0.72 (0.50–0.89)	6,559 (4,931–9,245)	0.11 (0.08–0.15)	−6.09^*^ (−6.24–−5.94)
Meningitis	7,954 (6,669–10,128)	0.12 (0.10–0.16)	3,434 (2,619–4,551)	0.06 (0.04–0.07)	−2.63^*^ (−2.99–−2.27)
Encephalitis	451 (332–523)	0.01 (0.01–0.01)	282 (212–363)	<0.01 (<0.01–0.01)	−1.47^*^ (−1.60–−1.33)
Sudden infant death syndrome	2,039 (887–3,504)	0.03 (0.01–0.06)	708 (371–1,049)	0.01 (0.01–0.02)	−3.30^*^ (−3.52–−3.09)

ASMR, age-standardized mortality rate; UI, uncertainty interval; AAPC, average annual percentage change; CI, confidential interval, *, *p* < 0.05.

The correlation analyses between SDI and both ASRs for all causes and the top five diseases are presented in Supplementary Figures S1, S2. Spearman analysis revealed a non-liner inverse relationship between SDI quintiles and ASRs. For ASMR, the correlation coefficients were *ρ* = −0.86 for all causes, *ρ* = −0.86 for COPD, *ρ* = −0.65 for IHD, *ρ* = −0.87 for LRI, *ρ* = −0.92 for neonatal disease, and *ρ* = −0.84 for stroke (all *p* < 0.05). For ASDR, the correlation coefficients were *ρ* = −0.88 for all causes, *ρ* = −0.86 for COPD, *ρ* = −0.69 for IHD, *ρ* = −0.91 for LRI, *ρ* = −0.92 for neonatal disease, and *ρ* = −0.85 for stroke (all *p* < 0.05). ASRs peaked in the low-SDI quintile and then gradually decreased, stabilizing at their lowest levels in the high-SDI quintile.

### 3.4 Global pattern of health burden attributable to PM2.5 from 1990 to 2021

**Figure 3:**
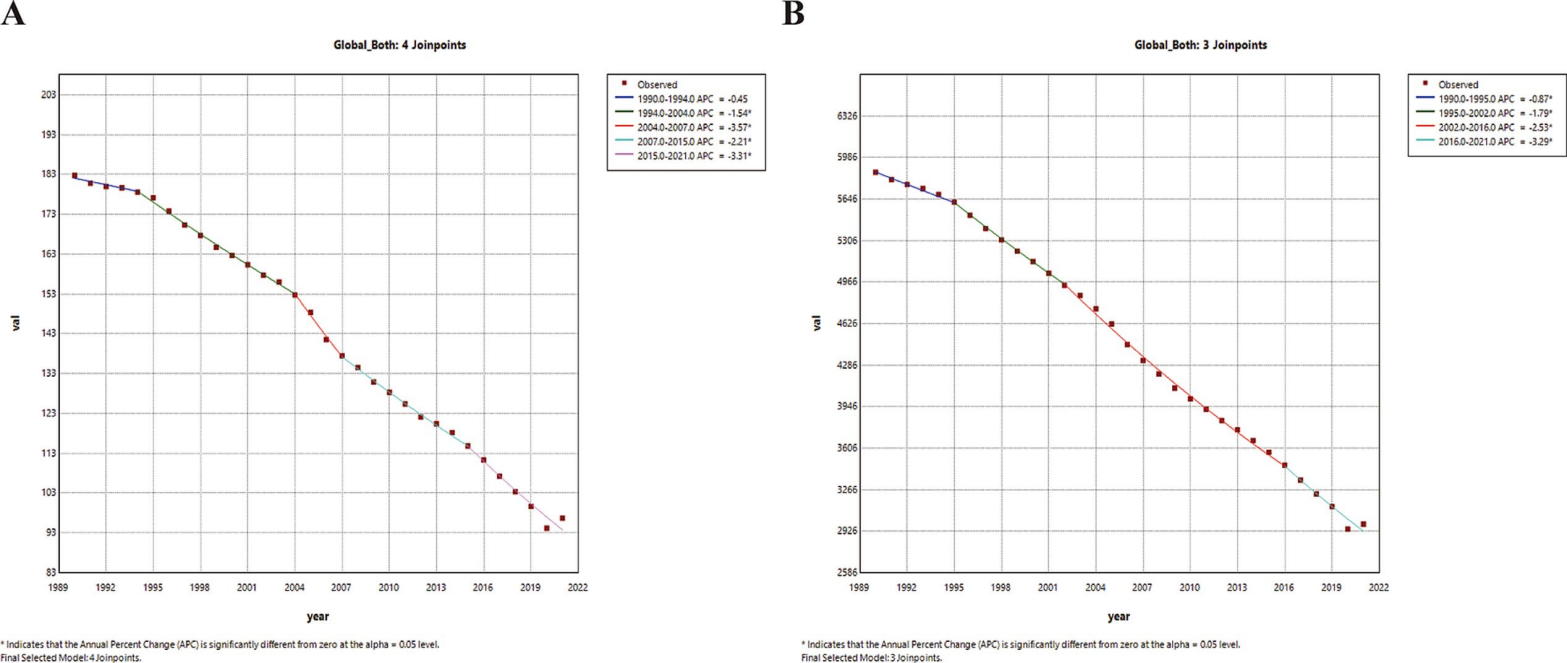
Temporal trends in global disease burden attributable to PM2.5 for both sexes from 1990 to 2021. **(A)** ASMR. **(B)** ASDR. ASMR, age-standardized mortality rates; ASDR, age-standardized DALY rates; APC, annual percentage change, ^*^, *p* < 0.05.

In 2021, the global number of deaths increased for both sexes, males and females when compared to 2019, while the number of DALYs decreased for both sexes, males and females. ASRs nearly halved compared to 1990 (Table 1). The AAPCs of ASMR for both sexes, females and males were −2.12 (95% CI: −2.40–1.83), −2.24 (−2.47–−2.02) and −1.97 (−2.16–−1.78), respectively. The AAPCs of ASDR for both sexes, females and males were −2.22 (−2.39–−2.05), −2.36 (−2.57–−2.16) and −2.12 (−2.37–−1.87), respectively. From a temporal perspective, the global ASMR for both sexes remained stable between 1990 and 1994 but declined significantly thereafter, while the global ASDR for both sexes decreased steadily from 1990 to 2021 (Figure 3).

### 3.5 Pattern of health burden attributable to PM2.5 across regions and countries from 1990 to 2021

The Joinpoint regression analysis and trends in ASRs for the SDI quintiles and seven super regions are shown in Supplementary Tables S2, S3 and Supplementary Figures S3, S4. All SDI quintiles and super regions experienced significant declines in both ASMR and ASDR from 2019 to 2021. Among the five SDI quintiles, the high SDI quintile saw the largest decrease in ASMR and ASDR, with AAPCs of −3.93 (95% CI: −4.09–−3.77) and −3.48 (−3.67–−3.29), respectively. The low SDI quintile showed the smallest reductions in ASMR and ASDR, with AAPCs of −0.95 (−1.13–−0.77) and −1.58 (−1.65–−1.50), respectively. Among the seven GBD super regions, the High-income GBD super region reported the most significant decreases in ASMR and ASDR, with AAPCs of −4.59 (−4.79–−4.38) and −4.08 (−4.20–−3.95), respectively. The lowest reduction in ASMR was observed in South Asia, with an AAPC of −1.02 (−1.62–−0.41), while Sub-Saharan Africa experienced the least decline in ASDR, with an AAPC of −1.64 (−1.68–−1.60).

Among the 204 countries and territories, only three countries -Lesotho, American Samoa, and Zimbabwe- experienced an increase in both ASDR and ASMR from 2019 to 2021. In contrast, more than 190 countries showed a significant decrease in both ASDR and ASMR, while a small number of countries exhibited stable trends. Lesotho had the largest increase in ASMR, with an AAPC of 0.64 (0.34–0.94). Meanwhile, Zimbabwe had the largest increase in ASDR, with an AAPC of 0.59 (0.29–0.88). The largest decrease of both ASMR and ASDR was recorded in Estonia, with AAPCs of −8.29 (−9.44–−7.13) and −7.90 (−8.70–−7.09) (Supplementary Table S4; Figures 2D,E).

### 3.6 The health burden of Level-3 diseases attributable to PM2.5 from 1990 to 2021

Among all Level-3 diseases, only DM saw a marked increase in both ASMR (0.14 [95% CI: 0.04–0.25]) and ASDR (0.90 [0.85–0.96]) globally from 1990 to 2021. In contrast, diarrheal diseases experienced the greatest decline in ASMR (−6.09 [−6.24–−5.94]) and ASDR (−6.07 [−6.22–−5.93]) (Tables 2; Supplementary Table S5).

**Figure 4:**
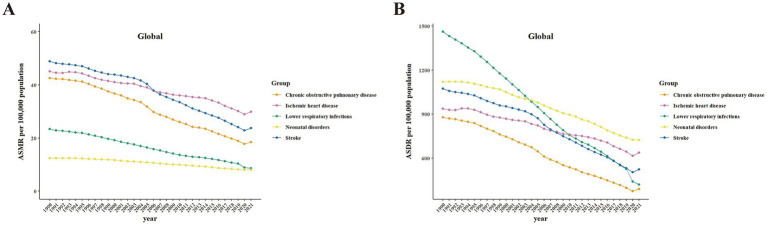
Temporal trends in health burden of the top five Level-3 diseases globally attributable to PM2.5 from 1990 to 2021. Data include both sexes. **(A)** ASMR. **(B)** ASDR. ASMR, age-standardized mortality rates; ASDR, age-standardized DALY rates.

The temporal trends of the top five diseases attributable to PM2.5 are presented in Figure 4 and Supplementary Figures S5, S6 in detail for the global, SDI quintiles, and seven GBD super regions, respectively. The detailed APCs and AAPCs are presented in Supplementary Tables S6, S7. From 1990 to 2021, the ASMR of IHD and COPD remained stable in the low SDI quintile, as did the ASMR of IHD in the low-middle SDI quintile. The ASMRs of all other diseases declined significantly across all SDI quintiles during this period. The ASDR for all diseases also declined significantly across all SDI quintiles from 1990 to 2021. While both ASDR and ASMR of Level-3 diseases declined significantly across seven GBD super regions, IHD remained stable in both ASMR and ASDR specifically in South Asia.

### 3.7 Projection for disease burdens attributable to PM2.5 from 2022 to 2036

**Figure 5:**
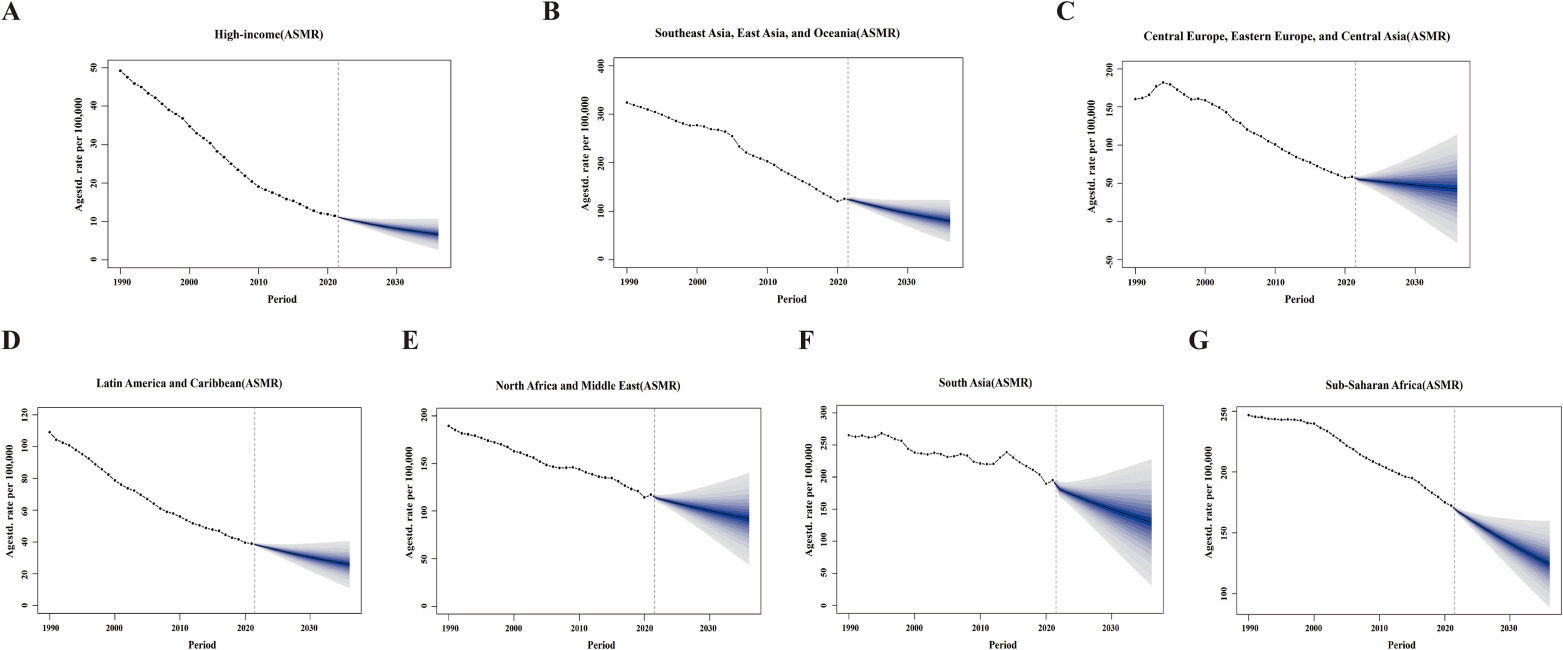
The projections of ASMR attributable to PM2.5 across seven GBD super regions from 2022 to 2036 using BAPC models. Data include both sexes. **(A)** High-income. **(B)** Southeast Asia, East Asia, and Oceania. **(C)** Central Europe, Eastern Europe, and Central Asia. **(D)** Latin America and the Caribbean. **(E)** North Africa and the Middle East. **(F)** South Asia. **(G)** Sub-Saharan Africa. Blue shades represent the corresponding confidence intervals of predictions between the 5 and 95% quantile with increments of 10%. Solid circles represent the observed number of cases. Solid lines represent the predictive means. Vertical dashed lines indicate the prediction start point. ASMR, age-standardized mortality rates; BAPC, Bayesian age-period-cohort.

Using comprehensive GBD data from 1990 to 2021, we applied the BAPC model to project both ASMR and ASDR for the seven super region disease burdens attributable to PM2.5 for both sexes from 2022 to 2036 (Figure 5; Supplementary Figure S7). The BAPC model predicted a decline in ASDR and ASMR across seven super regions over the next 15 years.

## 4 Discussion

This study evaluated the health burden attributable to PM2.5 across different ages, sexes, regions, countries and territories. We examined the trends in disease burden attributed to PM2.5 from 1990 to 2021 and forecasted this health burden for the next 15 years. Despite significant reductions in PM2.5-attributed health concerns over the past 32 years, it remained a critical risk factor with substantial impacts on global health. Key highlights include the identification of IHD and stroke as major contributors to the disease burden. Projections show a decline in ASRs across the seven super regions over the next 15 years. However, regions such as South Asia and sub-Saharan Africa are expected to remain disproportionately affected.

In 2021, PM2.5 exposure contributed to approximately 7.83 million deaths and 231.5 million DALYs. Significant disparities exist across SDI quintiles, GBD super regions, and individual countries. The low SDI quintile faced the highest ASMR and ASDR. Correlation analysis supported a significant negative correlation between the ASRs and SDI across different countries. South Asia and sub-Saharan Africa are the two GBD super regions with the highest disease burden attributable to PM2.5, while the High-income region experiences the lowest disease burden. Furthermore, among the countries with the highest ASMR and ASDR, all but Vanuatu—a country in the low-middle SDI quintile- are low SDI countries in sub-Saharan Africa. These findings highlight the strong association between poverty and disease burden tied to PM2.5 pollution. A substantial body of evidence consistently indicates that air pollution is one of the leading causes of mortality in LMICs (15, 16). These countries often have less stringent air quality regulations, widespread use of outdated and polluting machinery and vehicles, subsidies for fossil fuels, congested urban transportation systems, rapidly expanding industrial sectors, and agricultural practices that involve burning crop residue, all of which compound to elevated levels of air pollution (7, 8). Regions with poor housing conditions and temporary settlements often face persistent challenges from PM2.5 pollution, largely due to reliance on wood fires and kerosene cooking stoves (17, 18). The reduction of PM2.5 emissions has become a top priority for federal governments and international organizations worldwide. Effective solutions are available, such as sustainable transportation, clean cooking and heating technologies, energy-efficient products and buildings, low-emission power generators, and efficient urban waste management.

In 2021, China and India had the highest numbers of deaths and DALYs attributed to PM2.5. Both countries also made significant progress in reducing ASRs since 1990. However, the vast land mass and large populations continue to pose substantial challenges for controlling PM2.5 pollution. From 2005 to 2015, the Chinese government implemented stringent policies to control emissions from power plants, industries, and transportation. As a result, there was a notable reduction in PM2.5 emissions, including black carbon, organic carbon, and sulfur dioxide (19). The decrease in population-weighted exposure to PM2.5 was largely attributed to a decreased use of household solid fuels and an increased adoption of cleaner energy sources (20). In 2019, India launched the National Clean Air Programme (NCAP), which has since made positive progress (21). The overall PM2.5 concentration in India; however, remained significantly higher than the World Health Organization (WHO) guidelines (22). The mortality rate in 2019 attributed to ambient PM2.5 in India was higher than in 1990, but there was a significant decline in the mortality rate associated with household PM2.5 (23). In Africa, household particulate matter pollution remained a critical issue, with 84% of the population still relying on fossil fuels as their primary cooking source as of 2020 (24). Household air pollution is a significant contributor to ambient air pollution, with residential cooking accounting for approximately 12% of global PM2.5 emissions (25). Improvements in household particulate matter pollution could be effective in reducing mortality in regions like Asia and Africa.

The 2021 data indicated that disease burden from PM2.5 varied across different age groups. Infants and children (under 5 years old), and older adults experienced a higher disease burden. PM2.5 exposure is linked to child mortality for those under five through both prenatal and postnatal pathways (26). Maternal exposure during pregnancy can impair fetal development, leading to low birth weight, preterm birth, and complications due to placental and umbilical cord dysfunction, inflammation, and hypoxia (27–29). Postnatal exposure to PM2.5 further increases the risk of mortality by adversely affecting respiratory health (30, 31). Shiferaw et al. identified a correlation between a 10-unit increase in lifetime average PM2.5 concentration and a 2.29-fold rise in mortality under the age of five (32). Moreover, older adults are also at higher risk of the detrimental effects of PM2.5 exposure due to weakened immunity and cardiopulmonary function (33). Evidence indicated that prolonged exposure to particulate matter aggravated the decline in lung function and substantially increased the likelihood of cardiovascular and respiratory diseases, leading to increased rates of morbidity and mortality (34, 35). Given these challenges, health services dedicated to these sensitive, vulnerable populations remain a high priority.

Our study revealed significant sex disparities in the health impacts of PM2.5. In 2021, males experienced higher disease burdens from PM2.5 exposure compared to females globally, across all five SDI quintiles and seven super regions. Previous studies had shown inconsistent findings regarding the health effects of PM2.5 between the sexes. Shin et al. examined respiratory hospitalization and mortality rates related to air pollution and found that males had higher hospitalization rates due to PM2.5 exposure (36). Conversely, in urban Beijing, females were found to have a higher risk of developing respiratory diseases following PM2.5 exposure (37). In Japan, there was a significant association between PM2.5 exposure and asthma in females but not in males (38). The underlying mechanisms contributing to the sex differences remain unclear, but physiological factors (e.g., lung volume, hormone levels) and behavioral factors (e.g., smoking, occupational exposures) may be involved (39). Chen et al. revealed that long-term exposure to PM2.5 components, including black carbon, organic carbon, and nitrate, was associated with a greater mortality risk in males, possibly due to their smoking habits (40). When examining the AAPC between 2019 and 2021, we observed that both sexes experienced a significant decline in ASMR and ASDR. However, the reduction was more pronounced in females than in males. This suggests that while both sexes have benefited from improved air quality and healthcare interventions, females experienced a greater reduction in disease burden.

We further assessed the disease burden of 13 Level-3 diseases attributable to PM2.5 and conducted a detailed analysis of the temporal trends of the top five diseases across different regions. Cardiovascular diseases (CVDs), particularly IHD and stroke, are the leading causes of global mortality and contributors to disability (41). Although PM2.5 primarily enters the body through the respiratory system, its greatest risk lies in its contribution to CVDs by inducing oxidative stress, increasing endothelial permeability, impairing vasomotor function, and triggering macrophage polarization and apoptosis (42). In this study, IHD and stroke were also identified as the leading causes of disease burden attributed to PM2.5. Although both ASRs significantly decreased between 1990 and 2021, the absolute number of deaths and DALYs from IHD and stroke in 2021 was still higher than in 1990. Global IHD and stroke have seen substantial increases in incidence, mortality, and DALYs from 1990 to 2019 despite corresponding decreases in ASRs. These trends indicate that the growth in raw numbers is driven primarily by demographic factors such as population growth and aging (43). Nearly 80% of CVD deaths globally occurred in LMICs, where its associated risk factors and health burden continued to rise due to epidemiological transitions (44). In LMICs, rapid urbanization, industrialization, and increased vehicle emissions have deteriorated air quality, further exacerbating cardiovascular risks (45). The present study found no significant change in the ASMR of CVDs for low-middle and low SDI quintiles from 1990 to 2021. This emphasizes the urgent need to address air pollution as a risk factor, particularly in economically disadvantaged areas. Furthermore, the health risks associated with PM2.5 exposure can arise at levels below the current regulatory limits established by air quality standards in these regions, underscoring the inadequacy of these thresholds in protecting public health (43, 46). Policy interventions should aim at stricter emission regulation to help mitigate this risk in economically disadvantaged regions (47).

Among the 13 Level-3 diseases attributed to PM2.5, DM was the only condition that showed increases in both the absolute number of deaths and DALYs, as well as in both ASRs, from 1990 to 2021. Previous meta-analysis results indicated a statistically significant association between exposure to PM2.5 and the increased prevalence of DM (48, 49). Air pollution, particularly PM2.5, plays a significant role in the development and progression of DM through multiple mechanisms. PM2.5 exposure activates inflammatory pathways (JNK, NF-κB andTLR4), increasing CRP and IL-6 levels and triggering systemic inflammation, which disrupts adipose function, impairs hepatic glucose metabolism, and reduces muscle insulin sensitivity (48, 50). Additionally, PM2.5 induces oxidative stress, mitochondrial dysfunction, and β-cell impairment (51, 52). Xie et al. reported in 2021 that the burden of DM attributable to PM2.5 was most severe in the low SDI quintile. From 1990 to 2021, ASMR declined in high and high-middle SDI quintiles, remained stable in middle SDI quintile, and increased in low-middle SDI and low SDI quintiles (53). The impact of PM2.5 on DM varies geographically, disproportionately affecting less developed regions, including LMICs and areas with lower SDIs. It is expected that economic development and epidemiological transitions will amplify the influence of PM2.5 in non-communicable diseases, particularly DM (53, 54). Other contributing factors, such as population growth, aging, and rapid industrialization in lower SDI quintiles, may further intensify this burden. Given these risks, regular blood glucose monitoring, pharmacological interventions, and complication management in lower SDI populations are imperative in reducing the disease burden (55).

We used the R-packages, BAPC and INLA, to predict the disease burden trend over the next 15 years. This approach avoided the use of Markov chain Monte Carlo (MCMC) algorithms to reduce computational time and complex convergence concerns (56). In this study, we used the BAPC model to predict the trends in ASMR and ASDR across seven super regions over the next 15 years. Encouragingly, both rates were projected to decline; however, due to factors such as population growth and aging, the absolute number of deaths and DALYs attributable to PM2.5 might continue to rise (6, 43). Therefore, ongoing monitoring of the health burden associated with air pollution remains essential.

Limitations in this study were as follows. Firstly, the accuracy of GBD estimates is influenced by the quality of model predictions and prior research, with data gaps in conflict regions and more reliable monitoring in high-income areas (57). Secondly, the GBD 2021 risk factor studies did not account for COVID-19’s impact, despite evidence that air pollution worsens COVID-19-related morbidity and mortality (58, 59). Thirdly, PM2.5’s complex composition and varying toxicity, especially from sources like biomass burning, complicate its contribution to the disease burden. Understanding the specific components of PM2.5 could improve efforts to reduce pollution and enhance medical interventions (60, 61).

## 5 Conclusion

In conclusion, our study provides a comprehensive assessment of the global, regional, and national health burdens attributable to PM2.5 in 2021, while also analyzing trends from 1990 to 2021 and projecting future impacts through 2036. Despite a significant decline in global age-standardized mortality and DALY rates, challenges persist, especially in low- and middle-income countries where populations are growing and aging. Our findings emphasize the continued need for global efforts to reduce pollution and mitigate its health risks.

## Data Availability

The original contributions presented in the study are included in the article/Supplementary material, further inquiries can be directed to the corresponding authors.

## Footnotes

^1^ https://vizhub.healthdata.org/gbd-results/

## Supplementary material

SUPPLEMENTARY FIGURE S1 Correlations between ASMR and SDI of 204 countries and territories. **(A)** All causes. **(B)** Chronic obstructive pulmonary disease. **(C)** Ischemic heart disease. **(D)** Lower respiratory infections. **(E)** Neonatal disorders. **(F)** Stroke. ASMR, agestandardized mortality rates; SDI, socio-demographic index; *ρ*, spearman’s correlation coefficients; *, *p* < 0.05.

SUPPLEMENTARY FIGURE S2 Correlations between ASDR and SDI of 204 countries and territories. **(A)** All causes. **(B)** Chronic obstructive pulmonary disease. **(C)** Ischemic heart disease. **(D)** Lower respiratory infections. **(E)** Neonatal disorders. **(F)** Stroke. ASDR, age standardized DALY rates; SDI, socio-demographic index; *ρ*, spearman’s correlation coefficients; *, *p* < 0.05.

SUPPLEMENTARY FIGURE S3 Temporal trends of ASMR attributed to PM2.5 in SDI quintiles and GBD super regions from 1990 to 2021. Data include both sexes. **(A)** High SDI. **(B)** High-middle SDI. **(C)** Middle SDI. **(D)** Low-middle SDI. **(E)** Low SDI. **(F)** High-income. **(G)** Southeast Asia, East Asia, and Oceania. **(H)** Central Europe, Eastern Europe, and Central Asia. **(I)** Latin America and the Caribbean. **(J)** North Africa and the Middle East. **(K)** South Asia. **(L)** Sub-Saharan Africa. ASMR, age-standardized mortality rates; SDI, socio-demographic index; APC, annual percentage change, *, *p* < 0.05.

SUPPLEMENTARY FIGURE S4 Temporal trends of ASDR attributed to PM2.5 in SDI quintiles and GBD super regions from 1990 to 2021. Data include both sexes. **(A)** High SDI. **(B)** High-middle SDI. **(C)** Middle SDI. **(D)** Low-middle SDI. **(E)** Low SDI. **(F)** High-income. **(G)** Southeast Asia, East Asia, and Oceania. **(H)** Central Europe, Eastern Europe, and Central Asia. **(I)** Latin America and the Caribbean. **(J)** North Africa and the Middle East. **(K)** South Asia. **(L)** Sub-Saharan Africa. ASDR, age-standardized DALY rate; SDI, socio-demographic index; APC, annual percentage change, *, *p* < 00.05.

SUPPLEMENTARY FIGURE S5 Temporal trends of ASMR for five level-3 disease burdens attributable to PM2.5 across SDI quintiles and GBD super regions from 1990 to 2021. Data include both sexes. **(A)** High SDI. **(B)** High-middle SDI. **(C)** Middle SDI. **(D)** Low-middle SDI. **(E)** Low SDI. **(F)** High-income. **(G)** Southeast Asia, East Asia, and Oceania. **(H)** Central Europe, Eastern Europe, and Central Asia. **(I)** Latin America and the Caribbean. **(J)** North Africa and the Middle East. **(K)** South Asia. **(L)** Sub-Saharan Africa. ASMR, age standardized mortality rates; SDI, socio-demographic index.

SUPPLEMENTARY FIGURE S6 Temporal trends of ASDRs for five level-3 disease burdens attributable to PM2.5 across SDI quintiles and GBD super regions from 1990 to 2021. Data include both sexes. **(A)** High SDI. **(B)** High-middle SDI. **(C)** Middle SDI. **(D)** Low-middle SDI. **(E)** Low SDI. **(F)** High-income. **(G)** Southeast Asia, East Asia, and Oceania. **(H)** Central Europe, Eastern Europe, and Central Asia. **(I)** Latin America and the Caribbean. **(J)** North Africa and the Middle East. **(K)** South Asia. **(L)** Sub-Saharan Africa. ASDR, age standardized DALY rates; SDI, socio-demographic index.

SUPPLEMENTARY FIGURE S7 The projections of ASDR attributable to PM2.5 across GBD super regions from 2022 to 2036 using BAPC models. Data include both sexes. **(A)** High-income. **(B)** Southeast Asia, East Asia, and Oceania. **(C)** Central Europe, Eastern Europe, and Central Asia. **(D)** Latin America and the Caribbean. **(E)** North Africa and the Middle East. **(F)** South Asia. **(G)** Sub-Saharan Africa. Blue shades represent the corresponding confidence intervals of predictions between the 5% and 95% quantile with increments of 10%. Solid circles represent the observed number of cases. Solid lines represent the predictive means. Vertical dashed lines indicate the prediction start point. ASDR, age standardized DALY rates; BAPC, Bayesian age-period-cohort.
