# Supplementary figures and images for: Temporal trends of particulate matter pollution and its health burden, 1990–2021, with projections to 2036: a systematic analysis for the global burden of disease study 2021
Source: Front Public Health. 2025 Apr 16;13:1579716. doi: 10.3389/fpubh.2025.1579716 (PMC12041061; doi:10.3389/fpubh.2025.1579716)

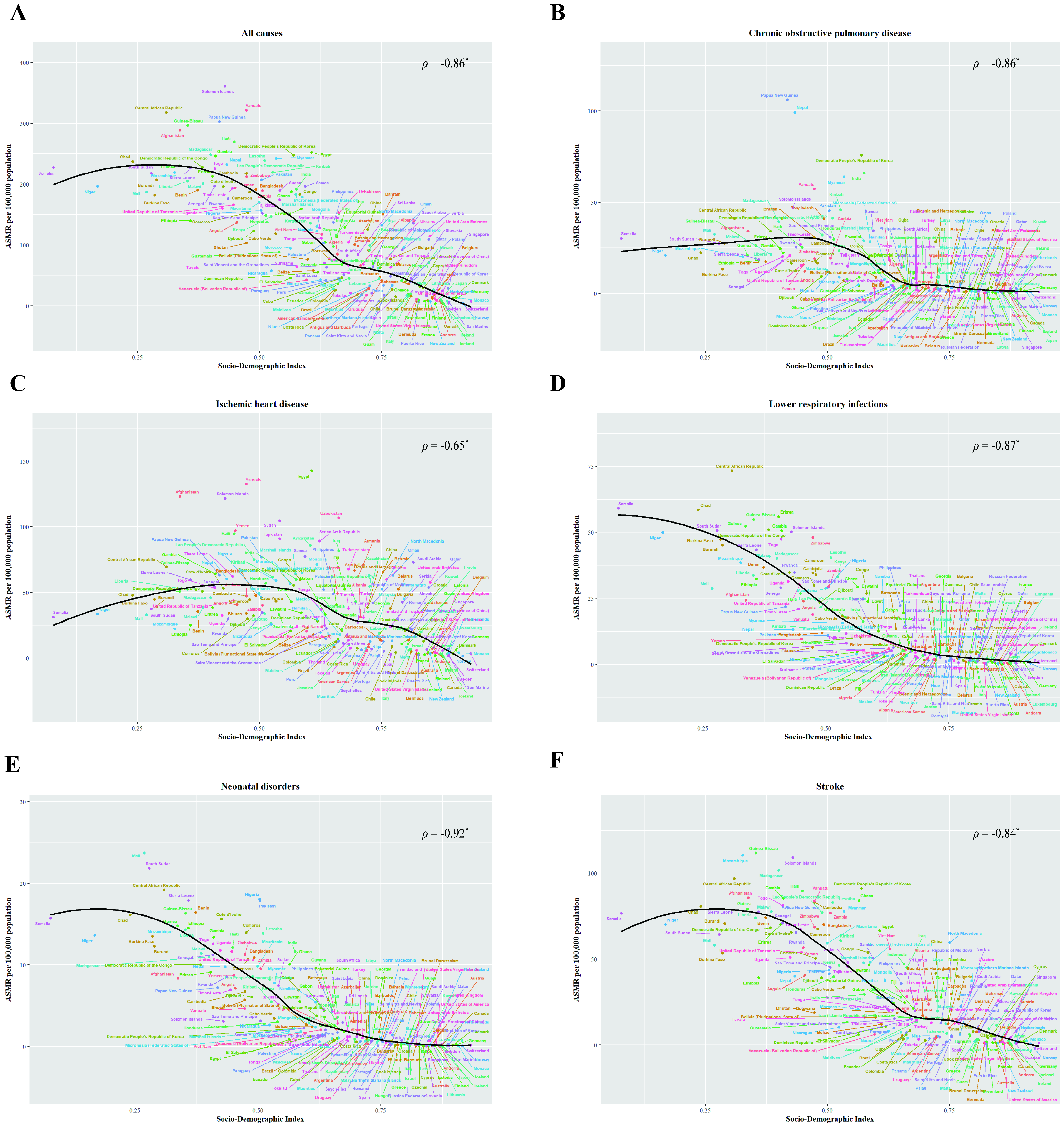

Supplement: SUPPLEMENTARY FIGURE S1 — Correlations between ASMR and SDI of 204 countries and territories. (A) All causes. (B) Chronic obstructive pulmonary disease. (C) Ischemic heart disease. (D) Lower respiratory infections. (E) Neonatal disorders. (F) Stroke. ASMR, agestandardized mortality rates; SDI, socio-demographic index; ρ, spearman’s correlation coefficients; *, p < 0.05. [file Image_1.TIF]

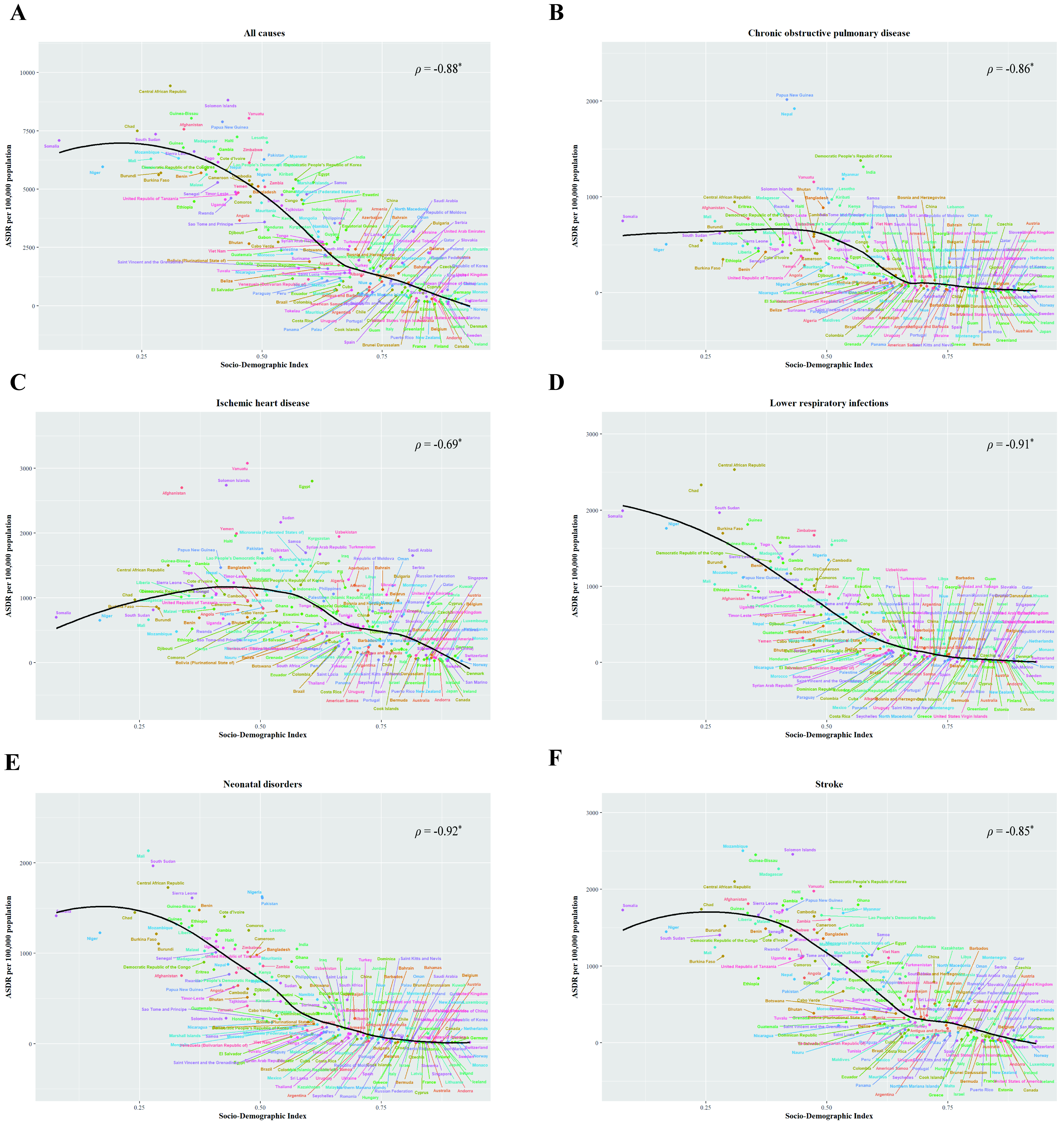

Supplement: SUPPLEMENTARY FIGURE S2 — Correlations between ASDR and SDI of 204 countries and territories. (A) All causes. (B) Chronic obstructive pulmonary disease. (C) Ischemic heart disease. (D) Lower respiratory infections. (E) Neonatal disorders. (F) Stroke. ASDR, age standardized DALY rates; SDI, socio-demographic index; ρ, spearman’s correlation coefficients; *, p < 0.05. [file Image_2.TIF]

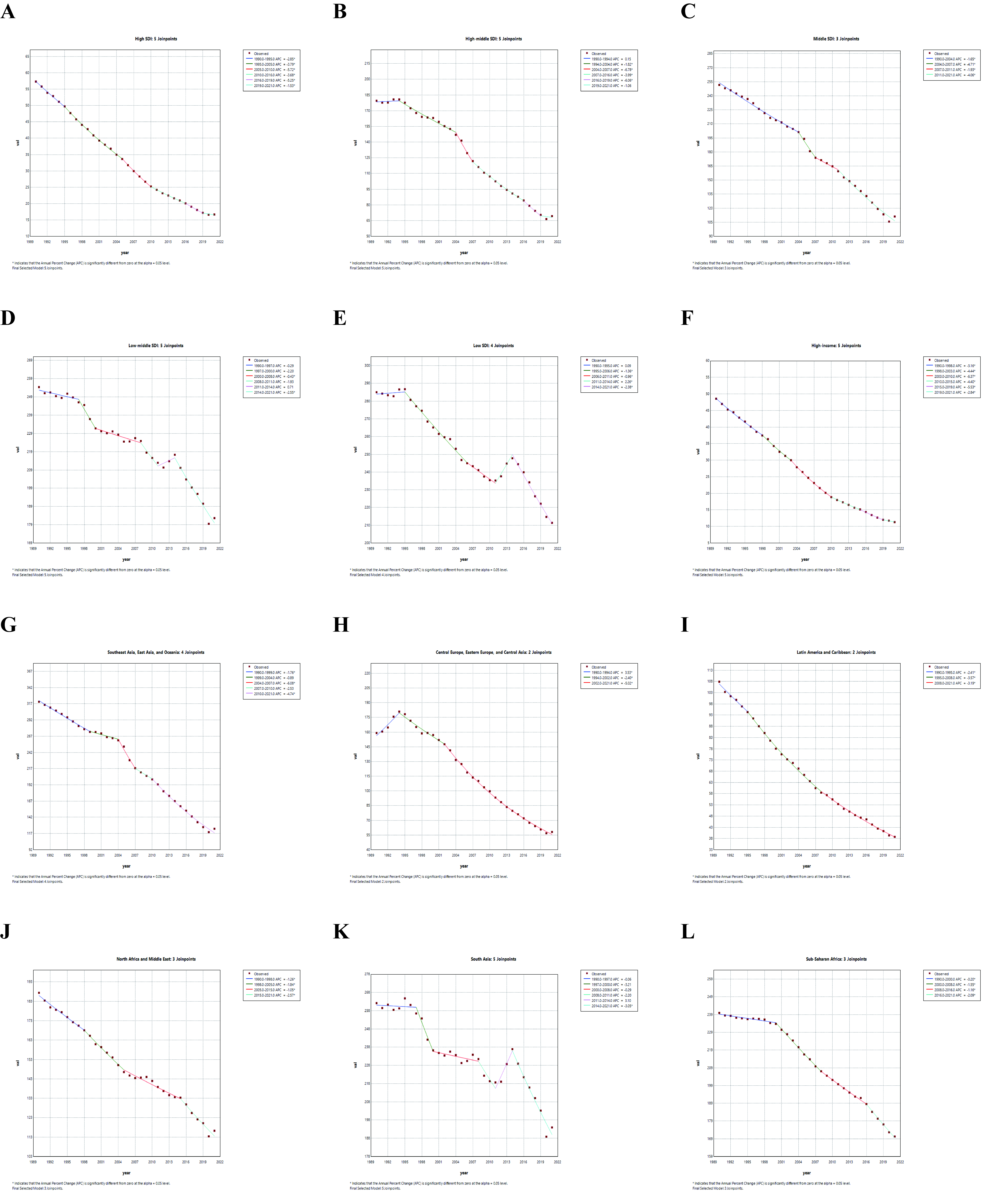

Supplement: SUPPLEMENTARY FIGURE S3 — Temporal trends of ASMR attributed to PM2.5 in SDI quintiles and GBD super regions from 1990 to 2021. Data include both sexes. (A) High SDI. (B) High-middle SDI. (C) Middle SDI. (D) Low-middle SDI. (E) Low SDI. (F) High-income. (G) Southeast Asia, East Asia, and Oceania. (H) Central Europe, Eastern Europe, and Central Asia. (I) Latin America and the Caribbean. (J) North Africa and the Middle East. (K) South Asia. (L) Sub-Saharan Africa. ASMR, age-standardized mortality rates; SDI, socio-demographic index; APC, annual percentage change, *, p < 0.05. [file Image_3.TIF]

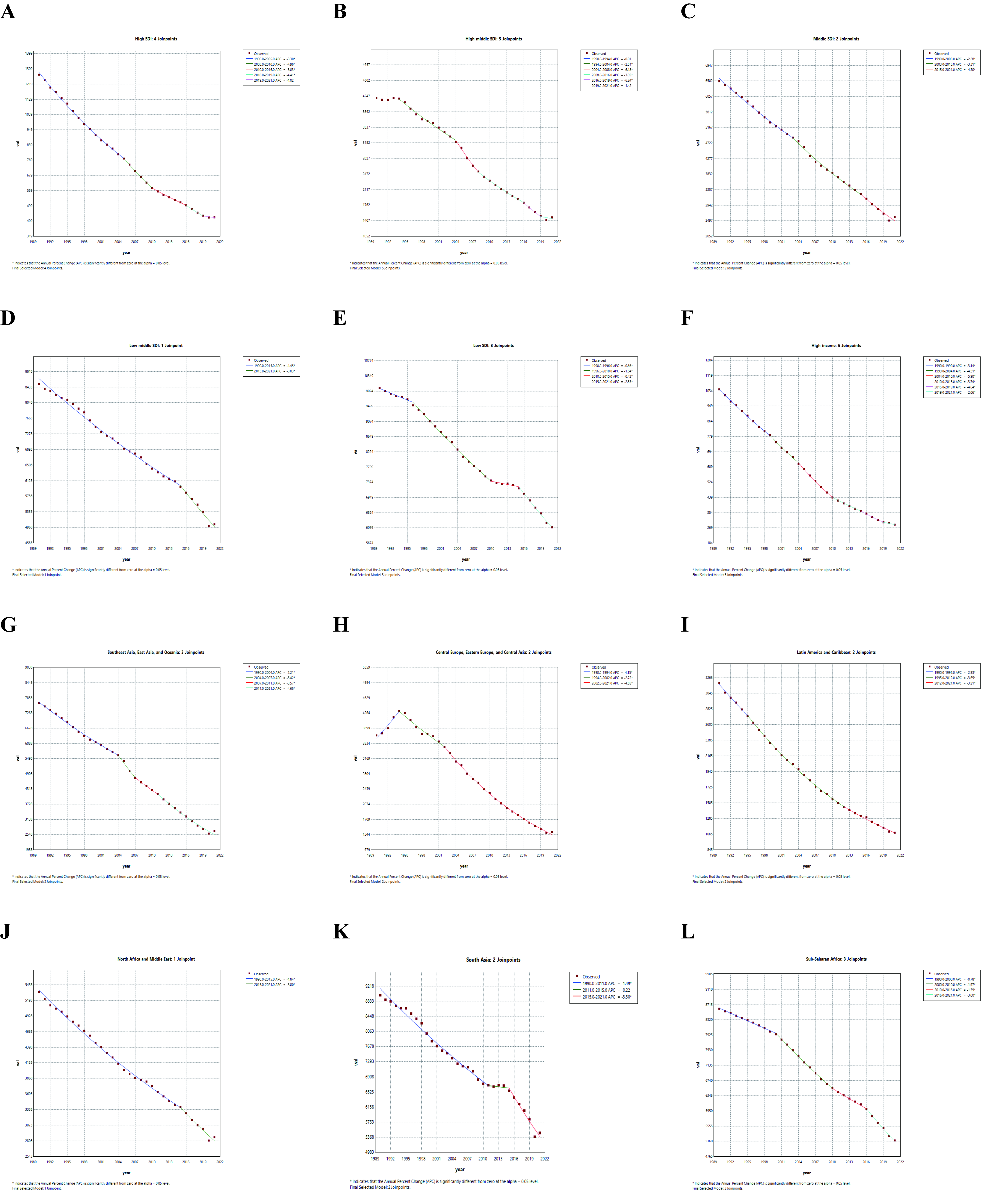

Supplement: SUPPLEMENTARY FIGURE S4 — Temporal trends of ASDR attributed to PM2.5 in SDI quintiles and GBD super regions from 1990 to 2021. Data include both sexes. (A) High SDI. (B) High-middle SDI. (C) Middle SDI. (D) Low-middle SDI. (E) Low SDI. (F) High-income. (G) Southeast Asia, East Asia, and Oceania. (H) Central Europe, Eastern Europe, and Central Asia. (I) Latin America and the Caribbean. (J) North Africa and the Middle East. (K) South Asia. (L) Sub-Saharan Africa. ASDR, age-standardized DALY rate; SDI, socio-demographic index; APC, annual percentage change, *, p < 00.05. [file Image_4.TIF]

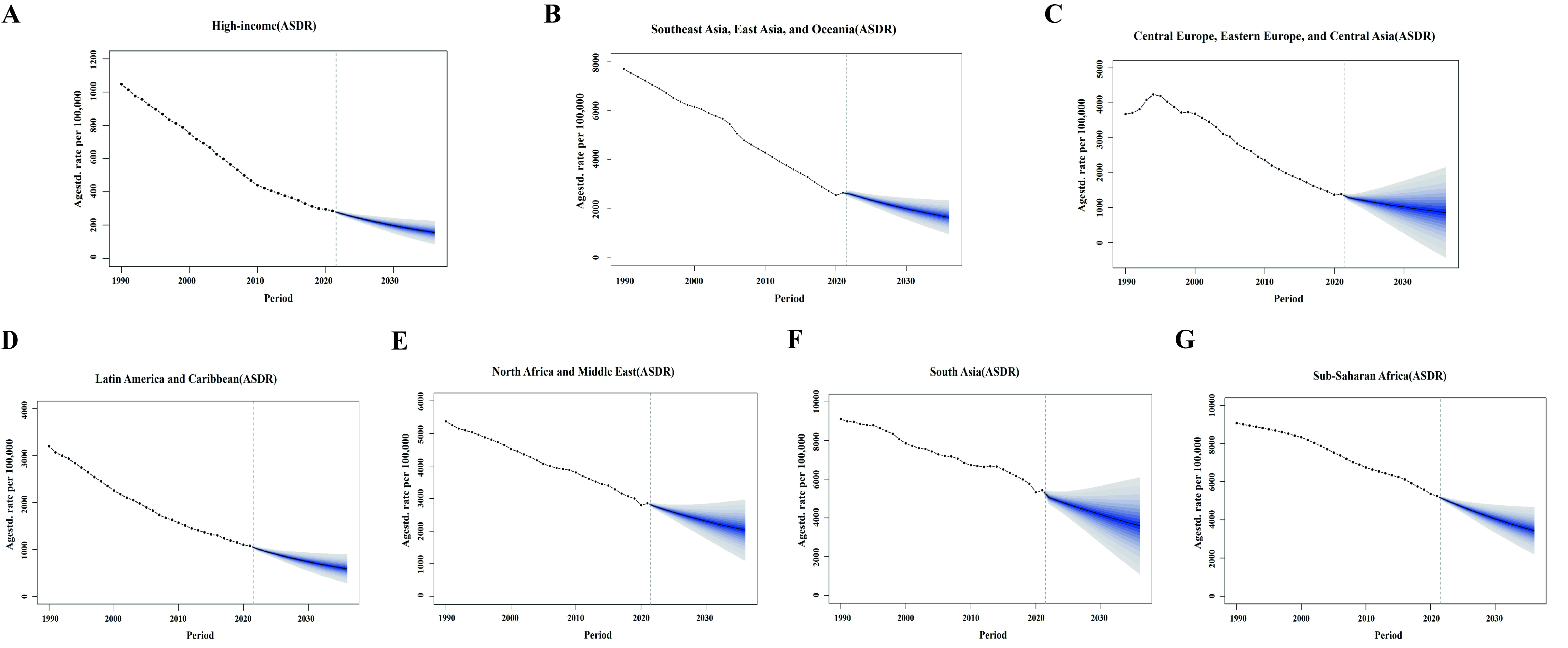

Supplement: SUPPLEMENTARY FIGURE S7 — The projections of ASDR attributable to PM2.5 across GBD super regions from 2022 to 2036 using BAPC models. Data include both sexes. (A) High-income. (B) Southeast Asia, East Asia, and Oceania. (C) Central Europe, Eastern Europe, and Central Asia. (D) Latin America and the Caribbean. (E) North Africa and the Middle East. (F) South Asia. (G) Sub-Saharan Africa. Blue shades represent the corresponding confidence intervals of predictions between the 5% and 95% quantile with increments of 10%. Solid circles represent the observed number of cases. Solid lines represent the predictive means. Vertical dashed lines indicate the prediction start point. ASDR, age standardized DALY rates; BAPC, Bayesian age-period-cohort. [file Image_7.TIF]
