# Supplementary material for: Temporal trends of particulate matter pollution and its health burden, 1990–2021, with projections to 2036: a systematic analysis for the global burden of disease study 2021
Source: Front Public Health. 2025 Apr 16;13:1579716. doi: 10.3389/fpubh.2025.1579716 (PMC12041061; doi:10.3389/fpubh.2025.1579716)
Supplement: Supplementary file 9 [file Table_2.DOCX]

| **Table S2. Deaths and ASMR attributable to PM2.5 in 1990 and 2021 and its average annual percentage change from 1990 to 2021, by location.** | | | | | | | | | | |  |  |
| --- | --- | --- | --- | --- | --- | --- | --- | --- | --- | --- | --- | --- |
| **Characteristics** | **1990** | | |  | | **2021** | |  | | **1990-2021** |  |  |
|  | **Deaths**  **n (95% UI)** | | **ASMR per 100,000**  **n (95% UI)** | | **Deaths**  **n (95% UI)** | | **ASMR per 100,000**  **n(95% UI)** | | **AAPC in ASMR**  **(95% CI)** | |  |  |
| **SDI region** |  |  | | |  | |  | |  | |  |  |
| **High SDI** |  |  | | |  | |  | |  | |  |  |
| Both | 628,074  (431,267-853,166) | 57.36  (39.46-77.68) | | | 383,145  (282,206-494,136) | | 16.66  (12.57-21.28) | | -3.93^*^  (-4.09 - -3.77) | |  |  |
| Female | 304,231  (208,300-414,633) | 44.12  (30.37-59.82) | | | 172,128  (121,088-224,527) | | 12.17  (8.87-15.65) | | -4.09^*^  (-4.22 - -3.95) | |  |  |
| Male | 323,843  (222,529-438,527) | 76.37  (52.20-103.62) | | | 211,017  (159,386-269,874) | | 22.17  (16.79-28.22) | | -3.94^*^  (-4.09 - -3.78) | |  |  |
| **High-middle SDI** |  |  | | |  | |  | |  | |  |  |
| Both | 1,545,409  (1,270,968-1,828,147) | 179.51  (146.41-212.58) | | | 1,327,461  (1,053,672-1,646,768) | | 69.25  (55.06-85.76) | | -3.06^*^  (-3.46 - -2.66) | |  |  |
| Female | 755,444  (606,906-912,957) | 147.47  (118.29-178.85) | | | 609,463  (468,667-771,949) | | 53.41  (41.27-67.43) | | -3.36^*^  (-3.64 - -3.08) | |  |  |
| Male | 789,965  (637,520,939,284) | 227.17  (184.77-269.23) | | | 717,998  (573,993-919,077) | | 91.34  (73.19-116.42) | | -2.94^*^  (-3.42 - -2.45) | |  |  |
| **Middle SDI** |  |  | | |  | |  | |  | |  |  |
| Both | 2,278,720  (1,955,652-2,595,307) | 251.62  (217.33-285.05) | | | 2,591,253  (2,065,517-3,185,581) | | 111.07  (88.84-136.20) | | -2.77^*^  (-3.15 - -2.38) | |  |  |
| Female | 1,054,811  (883,898-1,219,042) | 223.90  (190.06-258.99) | | | 1,114,724  (871,925-1,371,433) | | 87.74  (68.69-107.45) | | -3.14^*^  (-3.59 - -2.70) | |  |  |
| Male | 1,223,909  (1,037,871-1,407,798) | 283.26  (242.61-326.59) | | | 1,476,529  (1,184,574-1,858,016) | | 140.24  (112.13-175.93) | | -2.31^*^  (-2.66 - -1.96) | |  |  |
| **Low-middle SDI** |  |  | | |  | |  | |  | |  |  |
| Both | 1,830,924  (1,483,692-2,119,913) | 254.35  (215.24-291.93) | | | 2,374,836  (2,010,869-2,697,815) | | 182.63  (153.24-207.76) | | -1.09^*^  (-1.52 - -0.65) | |  |  |
| Female | 823,507  (628,976-975,224) | 230.57  (187.31-269.49) | | | 1,066,388  (873,268-1,239,804) | | 157.42  (128.53-183.78) | | -1.23^*^  (-1.58 - -0.88) | |  |  |
| Male | 1,007,417  (832,330-1,165,064) | 277.67  (233.93-317.26) | | | 1,308,448  (1,103,457-1,497,768) | | 210.92  (177.72-241.62) | | -0.98^*^  (-1.10 - -0.85) | |  |  |
| **Low SDI** |  |  | | |  | |  | |  | |  |  |
| Both | 960,370  (665,638-1,201,443) | 285.03  (222.46-333.36) | | | 1,150,978  (925,885-1,364,945) | | 211.39  (174.87-245.86) | | -0.95^*^  (-1.13 - -0.77) | |  |  |
| Female | 441,362  (297,177-560,293) | 262.35  (204.49-312.19) | | | 513,268  (407,209-615,221) | | 188.93  (152.64-222.80) | | -1.05^*^  (-1.31 - -0.80) | |  |  |
| Male | 519,008  (376,856-642,817) | 307.71  (246.22-365.27) | | | 637,711  (523,440-751,972) | | 235.53  (198.06-272.35) | | -0.85^*^  (-1.01 - -0.70) | |  |  |
| **GBD super region** |  |  | | |  | |  | |  | |  |  |
| **High-income** |  |  | | |  | |  | |  | |  |  |
| Both | 579,727  (370,849-826,331) | 48.57  (31.11-69.04) | | | 286,837  (192,866-386,374) | | 11.31  (7.75-15.16) | | -4.59^*^  (-4.79 - -4.38) | |  |  |
| Female | 279,285  (172,460-403,901) | 36.93  (23.07-52.96) | | | 132,488  (86,958-182,107) | | 8.30  (5.61-11.23) | | -4.70^*^  (-4.87 - -4.52) | |  |  |
| Male | 300,441  (192,128-423,313) | 65.43  (41.84-92.44) | | | 154,349  (106,814-206,317) | | 15.12  (10.50-20.18) | | -4.62^*^  (-4.84 - -4.40) | |  |  |
| **Southeast Asia, East Asia, and Oceania** |  |  | | |  | |  | |  | |  |  |
| Both | 2,861,817  (2,470,836-3,271,698) | 320.09  (275.97-362.71) | | | 3,015,190  (2,399,021-3,778,219) | | 124.42  (98.63-154.83) | | -3.18^*^  (-3.65 - -2.71) | |  |  |
| Female | 1,349,094  (1,134,777-1,572,540) | 281.51  (237.79-327.33) | | | 1,311,333  (996,874,1,650,421) | | 97.20  (74.48-121.98) | | -3.43^*^  (-3.77 - -3.10) | |  |  |
| Male | 1,512,723  (1,266,549-1,777,043) | 372.09  (316.03-430.46) | | | 1,703,857  (1,330,220-2,192,189) | | 161.63  (127.05-205.12) | | -2.85^*^  (-3.27 - -2.42) | |  |  |
| **Central Europe, Eastern Europe, and Central Asia** |  |  | | |  | |  | |  | |  |  |
| Both | 666,982  (457,064-899,931) | 159.12  (109.31-214.68) | | | 372,483  (276,222-490,846) | | 58.24  (43.55-76.27) | | -3.28^*^  (-3.54 - -3.02) | |  |  |
| Female | 355,305  (239,962-478,197) | 129.21  (87.86-173.88) | | | 195,045  (142,567-260,673) | | 46.29  (34.3-61.48) | | -3.40^*^  (-3.69 - -3.11) | |  |  |
| Male | 311,677  (218,320-416,537) | 209.54  (145.89-279.08) | | | 177,438  (133,608-230,463) | | 75.46  (56.69-97.83) | | -3.29^*^  (-3.54 - -3.03) | |  |  |
| **Latin America and Caribbean** |  |  | | |  | |  | |  | |  |  |
| Both | 244,729  (181,573-310,034) | 107.89  (79.66-136.41) | | | 229,263  (166,648-294,936) | | 38.78  (28.15-49.90) | | -3.22^*^  (-3.34 - -3.10) | |  |  |
| Female | 114,395  (85,878-142,910) | 98.13  (73.20-122.83) | | | 110,347  (79,125-142,246) | | 33.55  (24.18-43.1) | | -3.37^*^  (-3.67 - -3.07) | |  |  |
| Male | 130,334  (96,138-165,816) | 118.31  (86.57-151.82) | | | 118,917  (86,678-153,474) | | 45.08  (32.89-58.22) | | -3.07^*^  (-3.18 - -2.96) | |  |  |
| **North Africa and Middle East** |  |  | | |  | |  | |  | |  |  |
| Both | 333,735  (271,291-398,620) | 187.38  (154.5-220.36) | | | 453,957  (370,872-538,108) | | 116.25  (95.01-136.53) | | -1.58^*^  (-1.76 - -1.40) | |  |  |
| Female | 149,824  (119,853-183,470) | 172.90  (141.53-202.75) | | | 198,170  (160,266-235,092) | | 105.21  (85.36-124.4) | | -1.62^*^  (-1.78 - -1.46) | |  |  |
| Male | 183,910  (150,911-217,673) | 200.97  (165.6-237.65) | | | 255,787  (208,756-303,863) | | 126.88  (104.04-149.66) | | -1.53^*^  (-1.71 - -1.35) | |  |  |
| **South Asia** |  |  | | |  | |  | |  | |  |  |
| Both | 1,748,164  (1,429,078-2,010,952) | 262.18  (220.22-300.55) | | | 2,525,171  (2,136,170-2,868,315) | | 193.96  (163.84-221.42) | | -1.02^*^  (-1.62 - -0.41) | |  |  |
| Female | 765,240  (584,893-904,288) | 233.31  (183.37-276.62) | | | 1,107,159  (896,076-1,287,641) | | 165.77  (133.74-193.21) | | -1.10^*^  (-1.70 - -0.49) | |  |  |
| Male | 982,924  (811,593-1,126,388) | 288.87  (241.83-334.02) | | | 1,418,012  (1,209,435-1,642,636) | | 225.24  (192.35-261.29) | | -0.95^*^  (-1.41 - -0.50) | |  |  |
| **Sub-Saharan Africa** |  |  | | |  | |  | |  | |  |  |
| Both | 815,058  (548,959-1,038,687) | 239.98  (179.20-289.16) | | | 950,319  (743,261-1,162,626) | | 170.27  (136.87-204.29) | | -1.10^*^  (-1.15 - -1.06) | |  |  |
| Female | 369,391  (239,309-479,726) | 214.32  (158.95-261.43) | | | 424,072  (325,772-525,625) | | 148.87  (116.41-181.79) | | -1.16^*^  (-1.22 - -1.10) | |  |  |
| Male | 445,667  (314,315-569,311) | 267.93  (201.97-323.09) | | | 526,247  (417,393-637,703) | | 195.46  (158.44-232.47) | | -1.01^*^  (-1.07 - -0.95) | |  |  |
| ASMR, age-standardized mortality rate; UI, uncertainty interval; AAPC, average annual percentage change; CI, confidential interval,^*^,*P* <0.05. | | | | | | | | | | |  | 3.85 (2.3 ,5.5) |
