# Supplementary material for: Temporal trends of particulate matter pollution and its health burden, 1990–2021, with projections to 2036: a systematic analysis for the global burden of disease study 2021
Source: Front Public Health. 2025 Apr 16;13:1579716. doi: 10.3389/fpubh.2025.1579716 (PMC12041061; doi:10.3389/fpubh.2025.1579716)
Supplement: Supplementary file 10 [file Table_3.DOCX]

| **Table S3**. **DALYs and ASDR attributable to PM2.5 in 1990 and 2021, and its average annual percentage change from 1990 to 2021, by location.** | | | | | | | |  |  |
| --- | --- | --- | --- | --- | --- | --- | --- | --- | --- |
| **Characteristics** | **1990** | |  | **2021** | |  | **1990-2021** |  |  |
|  | **DALYs**  **n (95% UI)** | **ASDR per 100,000**  **n (95% UI)** | **DALYs**  **n (95% UI)** | | **ASDR per 100,000**  **n(95% UI)** | | **AAPC in ASDR**  **(95% CI)** |  |  |
| **SDI region** |  |  |  | |  | |  |  |  |
| **High SDI** |  |  |  | |  | |  |  |  |
| Both | 13,352,746  (9,438,088-17,610,835) | 1,275.07  (910.16-1,668.60) | 8,236,387  (6,239,929-10,398,103) | | 431.38  (334.22-537.24) | | -3.48^*^  (-3.67 - -3.29) |  |  |
| Female | 5,717,157  (4,029,955-7,557,273) | 931.45  (672.29-1209.44) | 3,353,146  (2,454,909-4,288,891) | | 311.44  (236.17-393.78) | | -3.48^*^  (-3.63 - -3.32) |  |  |
| Male | 7,635,589  (5,349,388-10,166,596) | 1706.69  (1,199.13-2,254.39) | 4,883,241  (3,743,520-6,158,244) | | 563.70  (436.14-705.18) | | -3.56^*^  (-3.76 - -3.35) |  |  |
| **High-middle SDI** |  |  |  | |  | |  |  |  |
| Both | 39,562,456  (32,894,449-46,737,496) | 4,204.32  (3,481.76-4,975.45) | 27,643,307  (22,477,547-34,211,203) | | 1481.88  (1,216.13-1,815.40) | | -3.34^*^  (-3.67 - -3.02) |  |  |
| Female | 17,526,235  (14,288,971-21,034,414) | 3,365.62  (2,725.49-4,051.89) | 11,746,233  (9,391,188-14,634,581) | | 1,124.90  (908.79-1,384.57) | | -3.60^*^  (-3.87 - -3.33) |  |  |
| Male | 22,036,222  (17,981,273-26,184,966) | 5,272.62  (4,329.26-6252.00) | 15,897,075  (12,911,406-20,362,756) | | 1,915.46  (1,564.60-2,422.03) | | -3.26^*^  (-3.66 - -2.86) |  |  |
| **Middle SDI** |  |  |  | |  | |  |  |  |
| Both | 81,484,628  (64,637,634-97,016,242) | 6,498.81  (5,455.31-7,466.23) | 63,574,457  (52,026,702-76,406,831) | | 2,605.44  (2,142.77-3,121.59) | | -3.08^*^  (-3.25 - -2.90) |  |  |
| Female | 35,992,714  (28,054,970-43,334,980) | 5,730.22  (4,716.98-6,727.51) | 26,362,581  (21,459,142-31,560,353) | | 2,078.13  (1,714.33-2,477.49) | | -3.33^*^  (-3.43 - -3.22) |  |  |
| Male | 45,491,914  (36,301,460-53,730,438) | 7,300.46  (6,100.95-8,425.61) | 37,211,876  (30,519,739-46,030,589) | | 3,198.59  (2,639.17-3,911.50) | | -2.79^*^  (-3.11 - -2.47) |  |  |
| **Low-middle SDI** |  |  |  | |  | |  |  |  |
| Both | 95,426,824  (70,303,340-115,552,090) | 8,510.87  (6,868.02-9,918.36) | 77,433,512  (64,788,178-90,113,245) | | 5,046.41  (4,254.45-5,819.22) | | -1.76^*^  (-1.85 - -1.66) |  |  |
| Female | 43,591,072  (30,131,463-54,000,669) | 7,820.58  (5,993.06-9,297.22) | 33,950,269  (27,998,741-40,075,854) | | 4,359.95  (3,594.38-5,126.87) | | -1.97^*^  (-2.10 - -1.85) |  |  |
| Male | 51,835,752  (40,007,357-62,023,456) | 9,174.12  (7,562.81-10,616.09) | 43,483,243  (36,867,219-50,644,263) | | 5,774.40  (4,925.03-6,629.99) | | -1.57^*^  (-1.66 - -1.49) |  |  |
| **Low SDI** |  |  |  | |  | |  |  |  |
| Both | 59,313,476  (38,470,947-78,007,376) | 10,003.12  (7,172.85-12,394.84) | 54,461,666  (42,615,605-66,989,287) | | 6,114.26  (5,006.33-7,220.39) | | -1.58^*^  (-1.65 - -1.50) |  |  |
| Female | 27,589,331  (16,711,777-36,741,970) | 9,356.75  (6,498.92-11,814.08) | 23,562,659  (18,086,685-29,072,076) | | 5,383.63  (4,302.75-6,447.57) | | -1.77^*^  (-1.90 - -1.65) |  |  |
| Male | 31,724,145  (21,548,319-41,461,838) | 10,625.77  (7,900.32-13,027.08) | 30,899,007  (24,373,888-37,885,451) | | 6,856.06  (5,697.96-8,041.06) | | -1.42^*^  (-1.51 - -1.33) |  |  |
| **GBD super region** |  |  |  | |  | |  |  |  |
| **High-income** |  |  |  | |  | |  |  |  |
| Both | 11,846,932  (7,769,851-16,545,982) | 1,041.91  (691.35-1,442.74) | 5,855,741  (4,052,248-7,886,667) | | 286.27  (201.50-381.66) | | -4.08^*^  (-4.20 - -3.95) |  |  |
| Female | 5,039,908  (3,295,976-7,105,133) | 749.78  (506.18-1,036.20) | 2,471,721  (1,665,980-3,361,184) | | 211.78  (147.73-283.91) | | -3.99^*^  (-4.13 - -3.84) |  |  |
| Male | 6,807,024  (4,463,402-9,471,916) | 1,415.32  (934.19-1,962.90) | 3,384,020  (2,346,504-4,538,223) | | 371.96  (260.55-493.27) | | -4.22^*^  (-4.43 - -4.00) |  |  |
| **Southeast Asia, East Asia, and Oceania** |  |  |  | |  | |  |  |  |
| Both | 91,984,982  (73,261,133-108,211,145) | 7,653.86  (6,444.80-8,850.14) | 67,610,108  (54,805,053-83,837,513) | | 2,687.83  (2,186.43-3,280.35) | | -3.50^*^  (-3.85 - -3.15) |  |  |
| Female | 41,298,784  (32,196,655-49,514,709) | 6,771.99  (5,484.69-7,978.59) | 28,144,724  (22,365,607-34,458,571) | | 2,124.49  (1,704.87-2,575.07) | | -3.77^*^  (-3.98 - -3.55) |  |  |
| Male | 50,686,198  (40731084,60073860) | 8,665.07  (7,298.55-10,097.65) | 39,465,384  (30,982,321-50,648,213) | | 3,349.90  (2,667.67-4,216.87) | | -3.11^*^  (-3.34 - -2.88) |  |  |
| **Central Europe, Eastern Europe, and Central Asia** |  |  |  | |  | |  |  |  |
| Both | 16,077,895  (11,493,791-20,978,525) | 3,729.12  (2,665.94-4,843.60) | 8,046,694  (6,136,979-10,355,523) | | 1,399.28  (1,071.90-1,795.09) | | -3.18^*^  (-3.40 - -2.96) |  |  |
| Female | 7,375,849  (5,197,121-9,714,107) | 2,838.21  (2,001.64-3,694.78) | 3,653,873  (2,750,283-4,761,757) | | 1,048.21  (796.10-1,344.95) | | -3.27^*^  (-3.52 - -3.02) |  |  |
| Male | 8,702,046  (6,250,676-11,466,766) | 4,998.02  (3,599.76-6,546.97) | 4,392,821  (3,363,568-5,652,539) | | 1,850.16  (1,425.40-2,374.60) | | -3.19^*^  (-3.42 - -2.96) |  |  |
| **Latin America and Caribbean** |  |  |  | |  | |  |  |  |
| Both | 10,195,572  (7,435,863-12,889,639) | 3,187.56  (2,387.21-3,982.11) | 6,367,563  (4,776,032-8,067,267) | | 1,084.10  (816.62-1,366.42) | | -3.41^*^  (-3.51 - -3.31) |  |  |
| Female | 4,585,860  (3,333,096-5,809,217) | 2,838.82  (2,127.74-3,529.00) | 2,940,665  (2,214,908-3,713,044) | | 939.72  (710.34-1,179.54) | | -3.50^*^  (-3.70 - -3.29) |  |  |
| Male | 5,609,712  (4,084,203-7,099,246) | 3,550.26  (2,652.68-4,477.34) | 3,426,898  (2,601,313-4,356,538) | | 1,247.63  (950.21-1,583.26) | | -3.35^*^  (-3.49 - -3.21) |  |  |
| **North Africa and Middle East** |  |  |  | |  | |  |  |  |
| Both | 14,994,360  (11,287,110-19,027,795) | 5,333.31  (4,339.59-6,381.14) | 13,607,276  (11,124,473-15,977,676) | | 2,871.13  (2,365.27-3,385.30) | | -2.07^*^  (-2.17 - -1.97) |  |  |
| Female | 6,629,605  (4,649,985-8,761,737) | 4,797.19  (3,835.17-5,883.24) | 5,701,614  (4,641,522-6,762,282) | | 2,503.21  (2,062.12-2,954.23) | | -2.14^*^  (-2.24 - -2.03) |  |  |
| Male | 8,364,755  (6,497,333-10,471,928) | 5,839.84  (4,783.49-6,911.37) | 7,905,662  (6,466,056-9,371,544) | | 3,219.59  (2,657.52-3,799.01) | | -2.00^*^  (-2.09 - -1.90) |  |  |
| **South Asia** |  |  |  | |  | |  |  |  |
| Both | 92,551,627  (69,742,244-110,668,772) | 8,984.51  (7,294.60-10,413.94) | 80,560,949  (68,745,930-92,313,537) | | 5,480.42  (4,694.75-6,270.19) | | -1.69^*^  (-1.95 - -1.43) |  |  |
| Female | 42,254,007  (29,674,646-52,007,834) | 8,279.69  (6,329.84-9,785.76) | 35,044,630  (29,013,307-40,601,493) | | 4,785.07  (3,963.27-5,551.22) | | -1.88^*^  (-2.11 - -1.64) |  |  |
| Male | 50,297,620  (39,567,879-59,850,898) | 9,625.44  (7,920.67-11,035.18) | 45,516,319  (38,838,186-52,804,212) | | 6,204.10  (5,304.06-7,160.59) | | -1.52^*^  (-1.85 - -1.19) |  |  |
| **Sub-Saharan Africa** |  |  |  | |  | |  |  |  |
| Both | 51,702,900  (32,861,870-69,017,074) | 8,606.75  (5,926.31-10,816.47) | 49,462,901  (37,697,684-62,139,333) | | 5,182.71  (4,128.54-6,309.84) | | -1.64^*^  (-1.68 - -1.60) |  |  |
| Female | 23,326,921  (13,706,809-31,617,050) | 7,759.88  (5,096.43-9,964.45) | 21,089,812  (15,897,006-26,597,025) | | 4,433.76  (3,446.35-5,458.89) | | -1.81^*^  (-1.85 - -1.77) |  |  |
| Male | 28,375,979  (19,087,887-37,528,972) | 9,459.88  (6,768.98-11,939.9) | 28,373,089  (22,025,540-35,767,594) | | 5,990.53  (4,839.38-7,203.12) | | -1.48^*^  (-1.54 - -1.42) |  |  |
| DALYs, disability-adjusted life years; ASDR, age-standardized disability-adjusted life year rate; UI, uncertainty interval; AAPC, average annual percentage change; CI, confidential interval, ^*^,*P* <0.05. | | | | | | | |  | 3.85 (2.3 ,5.5) |
