# Supplementary material for: Temporal trends of particulate matter pollution and its health burden, 1990–2021, with projections to 2036: a systematic analysis for the global burden of disease study 2021
Source: Front Public Health. 2025 Apr 16;13:1579716. doi: 10.3389/fpubh.2025.1579716 (PMC12041061; doi:10.3389/fpubh.2025.1579716)
Supplement: Supplementary file 12 [file Table_5.DOCX]

| **Table S5. Global DALYs of diseases attributable to PM2.5 in 1990 and 2021, and the temporal trend from 1990 to 2021.** | | | | | | | | | |  |  |
| --- | --- | --- | --- | --- | --- | --- | --- | --- | --- | --- | --- |
| **Disease type** | **1990** | | |  | | **2021** | |  | **1990-2021** |  |  |
|  | **DALYs**  **n (95% UI)** | | **ASDR per 100,000**  **n (95% UI)** | | **DALYs**  **n (95% UI)** | | **ASDR per 100,000**  **n(95% UI)** | | **AAPC in ASDR**  **(95% CI)** |  |  |
| Ischemic heart disease | 36,568,812  (28,680,230-45,510,296) | 936.39  (729.98-1,165.82) | | | 54,675,670  (41,652,489-67,418,886) | | 638.48  (486.47-787.82) | | -1.31^*^  (-1.49 - -1.13) |  |  |
| Stroke | 42,304,117  (34,553,910-49,981,910) | 1,073.52  (877.41-1,276.32) | | | 44,962,167  (35,020,339-55,467,024) | | 523.30  (407.96-645.58) | | -2.36^*^  (-2.61 - -2.11) |  |  |
| Chronic obstructive  pulmonary disease | 33,509,790  (28,117,102-38,092,060) | 877.48  (736.13-995.54) | | | 33,238,712  (26,680,066-41,336,741) | | 389.50  (312.59-484.62) | | -2.67^*^  (-2.93 - -2.41) |  |  |
| Lower respiratory infections | 87,315,640  (20,780,746-139,682,971) | 1462.20  (340.59-2,338.31) | | | 29,098,331  (6,988,265-48,127,683) | | 420.09  (106.35-693.12) | | -4.01^*^  (-4.24 - -3.78) |  |  |
| Upper respiratory infections | 23,782  (6,478-48,148) | 0.37  (0.10-0.75) | | | 11,708  (2,800-28,082) | | 0.19  (0.05-0.45) | | -2.21^*^  (-2.27 - -2.15) |  |  |
| Otitis media | 1,363  (512-2,947) | 0.02  (0.01-0.05) | | | 327  (156-626) | | 0.01  (＜0.01-0.01) | | -4.44^*^  (-4.64 - -4.24) |  |  |
| Neonatal disorders | 71,710,489  (65,623,349-77,654,692) | 1,120.38  (1,025.34-1,213.25) | | | 44,737,311  (37,766,690-52,293,054) | | 723.06  (610.39-845.18) | | -1.42^*^  (-1.53 - -1.31) |  |  |
| Diabetes mellitus | 4,568,390  (2,676,365-6,604,454) | 112.67  (66.24-162.08) | | | 12,904,494  (7,501,414-19,485,254) | | 148.92  (86.50-224.91) | | 0.90^*^  (0.85 - 0.96) |  |  |
| Tracheal, bronchus,  and lung cancer | 6,863,711  (4,419,307-9,265,547) | 165.53  (106.63-223.80) | | | 8,934,120  (5,681,090-12,409,780) | | 102.08  (64.89-141.62) | | -1.60^*^  (-1.83 - -1.36) |  |  |
| Diarrheal diseases | 4,134,309  (2,908,927-5,122,753) | 64.76  (45.57-80.25) | | | 593,960  (446,774-835,347) | | 9.60  (7.22-13.50) | | -6.07^*^  (-6.22 - -5.93) |  |  |
| Meningitis | 715,796  (600,123-911,397) | 11.21  (9.40-14.27) | | | 309,052  (235,709-409,534) | | 4.99  (3.81-6.62) | | -2.63^*^  (-2.99 - -2.27) |  |  |
| Encephalitis | 40,616  (29,886-47,086) | 0.64  (0.47-0.74) | | | 25,343  (19,040-32,695) | | 0.41  (0.31-0.53) | | -1.47^*^  (-1.60 - -1.33) |  |  |
| Sudden infant death syndrome | 183,423  (79,813-315,237) | 2.88  (1.25-4.95) | | | 63,713  (33,411-94,401) | | 1.03  (0.54-1.52) | | -3.30^*^  (-3.52 - -3.09) |  |  |
| DALYs, disability-adjusted life years; ASDR, age-standardized disability-adjusted life year rate; UI, uncertainty interval; AAPC, average annual percentage change; CI, confidential interval; ^*^,*P* < 0.05. | | | | | | | | | |  | 3.85 (2.3 ,5.5) |
