# Supplementary material for: Temporal trends of particulate matter pollution and its health burden, 1990–2021, with projections to 2036: a systematic analysis for the global burden of disease study 2021
Source: Front Public Health. 2025 Apr 16;13:1579716. doi: 10.3389/fpubh.2025.1579716 (PMC12041061; doi:10.3389/fpubh.2025.1579716)
Supplement: Supplementary file 13 [file Table_6.DOCX]

| **Table S6. Joinpoint regression analysis: trends in ASMR of top five disease across global, five SDI quintiles, and seven GBD super regions** | | | |
| --- | --- | --- | --- |
| **Disease type** | **Period** | **APC(95%CI)** | **AAPC(95%CI)** |
| **Global** |  |  |  |
| Ischemic heart disease | 1990-1994 | -0.17 (-1.36 - 1.02) | -1.41^*^ (-1.60 - -1.22) |
|  | 1994-2015 | -1.28^*^ (-1.38 - -1.18) |  |
|  | 2015-2021 | -2.68^*^ (-3.30 - -2.06) |  |
| Stroke | 1990-2004 | -1.12^*^ (-1.20 - -1.05) | -2.37^*^ (-2.63 - -2.11) |
|  | 2004-2007 | -4.54^*^ (-6.07 - -3.00) |  |
|  | 2007-2016 | -3.03^*^ (-3.22 - -2.85) |  |
|  | 2016-2019 | -4.76^*^ (-6.57 - -2.92) |  |
|  | 2019-2021 | -1.10 (-3.12 - 0.97) |  |
| Chronic obstructive pulmonary disease | 1990-1995 | -0.60^*^ (-0.95 - -0.25) | -2.70^*^ (-3.00 - -2.41) |
|  | 1995-2004 | -2.32^*^ (-2.48 - -2.15) |  |
|  | 2004-2007 | -5.08^*^ (-6.66 - -3.47) |  |
|  | 2007-2016 | -2.98^*^ (-3.18 - -2.79) |  |
|  | 2016-2019 | -4.90^*^ (-6.96 - -2.79) |  |
|  | 2019-2021 | -1.40 (-3.90 - 1.16) |  |
| Lower respiratory infections | 1990-1996 | -1.25^*^ (-1.94 - -0.57) | -3.18^*^ (-3.41 - -2.94) |
|  | 1996-2018 | -3.06^*^ (-3.16 - -2.95) |  |
|  | 2018-2021 | -7.75^*^ (-9.72 - -5.74) |  |
| Neonatal disorders | 1990-1994 | -0.09 (-0.33 - 0.14) | -1.42^*^ (-1.53 - -1.31) |
|  | 1994-1999 | -0.95^*^ (-1.21 - -0.70) |  |
|  | 1999-2005 | -1.47^*^ (-1.65 - -1.28) |  |
|  | 2005-2014 | -1.71^*^ (-1.81 - -1.62) |  |
|  | 2014-2018 | -2.62^*^ (-3.17 - -2.06) |  |
|  | 2018-2021 | -1.40^*^ (-2.08 - -0.72) |  |
| **High SDI** |  |  |  |
| Ischemic heart disease | 1990-1994 | -3.35^*^ (-3.86 - -2.83) | -4.48^*^ (-4.66 - -4.29) |
|  | 1994-2005 | -4.67^*^ (-4.78 - -4.56) |  |
|  | 2005-2010 | -6.01^*^ (-6.42 - -5.61) |  |
|  | 2010-2016 | -4.47^*^ (-4.77 - -4.16) |  |
|  | 2016-2019 | -5.29^*^ (-6.67 - -3.89) |  |
|  | 2019-2021 | -0.48 (-2.05 - 1.12) |  |
| Stroke | 1990-2004 | -3.66^*^ (-3.75 - -3.57) | -4.14^*^ (-4.35 - -3.92) |
|  | 2004-2011 | -6.11^*^ (-6.38 - -5.83) |  |
|  | 2011-2016 | -3.10^*^ (-3.63 - -2.58) |  |
|  | 2016-2019 | -5.78^*^ (-7.46 - -4.07) |  |
|  | 2019-2021 | -0.50 (-2.41 - 1.45) |  |
| Chronic obstructive pulmonary disease | 1990-2003 | -2.41^*^ (-2.53 - -2.29) | -3.47^*^ (-3.79 - -3.15) |
|  | 2003-2012 | -5.01^*^ (-5.24 - -4.79) |  |
|  | 2012-2016 | -3.14^*^ (-4.28 - -1.98) |  |
|  | 2016-2019 | -5.91^*^ (-8.30 - -3.47) |  |
|  | 2019-2021 | -0.22 (-2.90 - 2.54) |  |
| Lower respiratory infections | 1990-1998 | -0.68^*^ (-1.23 - -0.12) | -3.38^*^ (-4.08 - -2.68) |
|  | 1998-2001 | -7.16^*^ (-11.79 - -2.28) |  |
|  | 2001-2011 | -3.65^*^ (-4.09 - -3.21) |  |
|  | 2011-2015 | -0.64 (-3.11 - 1.89) |  |
|  | 2015-2019 | -4.05^*^ (-6.52 - -1.53) |  |
|  | 2019-2021 | -10.65^*^ (-15.26 - -5.79) |  |
| Neonatal disorders | 1990-1999 | -4.74^*^ (-5.02 - -4.47) | -4.89^*^ (-5.14 - -4.65) |
|  | 1999-2006 | -3.52^*^ (-4.06 - -2.98) |  |
|  | 2006-2011 | -6.50^*^ (-7.33 - -5.67) |  |
|  | 2011-2017 | -3.67^*^ (-4.25 - -3.08) |  |
|  | 2017-2021 | -7.39^*^ (-8.42 - -6.34) |  |
| **High-middle SDI** |  |  |  |
| Ischemic heart disease | 1990-1994 | 2.23^*^ (0.95 - 3.52) | -2.14^*^ (-2.65 - -1.62) |
|  | 1994-1997 | -2.78 (-6.57 - 1.17) |  |
|  | 1997-2003 | -0.88^*^ (-1.64 - -0.11) |  |
|  | 2003-2015 | -3.09^*^ (-3.32 - -2.86) |  |
|  | 2015-2019 | -5.29^*^ (-7.00 - -3.56) |  |
|  | 2019-2021 | -1.28 (-5.15 - 2.75) |  |
| Stroke | 1990-2004 | -1.07^*^ (-1.23 - -0.91) | -3.37^*^ (-3.84 - -2.89) |
|  | 2004-2008 | -7.38^*^ (-8.95 - -5.78) |  |
|  | 2008-2016 | -4.54^*^ (-5.00 - -4.07) |  |
|  | 2016-2019 | -6.94^*^ (-10.44 - -3.31) |  |
|  | 2019-2021 | -0.90 (-4.89 - 3.25) |  |
| Chronic obstructive pulmonary disease | 1990-1994 | -0.75^*^ (-1.40 - -0.11) | -4.79^*^ (-5.15 - -4.43) |
|  | 1994-2001 | -2.64^*^ (-3.00 - -2.27) |  |
|  | 2001-2004 | -4.79^*^ (-7.12 - -2.40) |  |
|  | 2004-2009 | -9.54^*^ (-10.39 - -8.67) |  |
|  | 2009-2019 | -6.08^*^ (-6.37 - -5.80) |  |
|  | 2019-2021 | -1.44 (-5.22 - 2.48) |  |
| Lower respiratory infections | 1990-1995 | -1.70^*^ (-2.73 - -0.67) | -4.02^*^ (-4.39 - -3.65) |
|  | 1995-2003 | -4.28^*^ (-4.88 - -3.67) |  |
|  | 2003-2009 | -6.16^*^ (-7.16 - -5.13) |  |
|  | 2009-2017 | -2.44^*^ (-3.08 - -1.80) |  |
|  | 2017-2021 | -6.21^*^ (-7.71 - -4.68) |  |
| Neonatal disorders | 1990-1995 | -1.72^*^ (-2.50 - -0.93) | -5.68^*^ (-5.98 - -5.38) |
|  | 1995-2001 | -4.45^*^ (-5.25 - -3.63) |  |
|  | 2001-2010 | -6.84^*^ (-7.19 - -6.48) |  |
|  | 2010-2014 | -5.24^*^ (-6.82 - -3.63) |  |
|  | 2014-2021 | -8.22^*^ (-8.71 - -7.71) |  |
| **Middle SDI** |  |  |  |
| Ischemic heart disease | 1990-2000 | -0.46^*^ (-0.75 - -0.17) | -0.92^*^ (-1.36 - -0.47) |
|  | 2000-2004 | 1.05 (-0.80 - 2.93) |  |
|  | 2004-2007 | -1.68 (-5.25 - 2.02) |  |
|  | 2007-2014 | -0.06 (-0.70 - 0.58) |  |
|  | 2014-2021 | -3.18^*^ (-3.73 - -2.64) |  |
| Stroke | 1990-2004 | -1.24^*^ (-1.42 - -1.06) | -2.96^*^ (-3.47 - -2.45) |
|  | 2004-2007 | -5.09^*^ (-8.69 - -1.36) |  |
|  | 2007-2010 | -2.10 (-5.95 - 1.90) |  |
|  | 2010-2021 | -4.75^*^ (-5.08 - -4.42) |  |
| Chronic obstructive pulmonary disease | 1990-1995 | -1.40^*^ (-2.18 - -0.62) | -4.43^*^ (-4.96 - -3.89) |
|  | 1995-2004 | -3.27^*^ (-3.63 - -2.90) |  |
|  | 2004-2007 | -7.82^*^ (-11.24 - -4.28) |  |
|  | 2007-2010 | -3.24 (-7.34 - 1.05) |  |
|  | 2010-2021 | -6.09^*^ (-6.51 - -5.67) |  |
| Lower respiratory infections | 1990-1995 | -2.99^*^ (-3.92 - -2.05) | -4.06^*^ (-4.35 - -3.76) |
|  | 1995-2007 | -4.29^*^ (-4.57 - -4.00) |  |
|  | 2007-2018 | -3.45^*^ (-3.80 - -3.10) |  |
|  | 2018-2021 | -7.07^*^ (-9.25 - -4.83) |  |
| Neonatal disorders | 1990-2000 | -1.95^*^ (-2.11 - -1.78) | -3.27^*^ (-3.38 - -3.15) |
|  | 2000-2014 | -3.24^*^ (-3.36 - -3.12) |  |
|  | 2014-2021 | -5.17^*^ (-5.58 - -4.75) |  |
| **Low-middle SDI** |  |  |  |
| Ischemic heart disease | 1990-1997 | 0.18 (-0.23 - 0.58) | -0.34 (-0.74 - 0.06) |
|  | 1997-2000 | -1.30 (-4.17 - 1.66) |  |
|  | 2000-2011 | 0.10 (-0.13 - 0.32) |  |
|  | 2011-2014 | 1.63 (-1.29 - 4.64) |  |
|  | 2014-2021 | -1.96^*^ (-2.36 - -1.56) |  |
| Stroke | 1990-1996 | -0.45 (-0.91 - 0.02) | -1.40^*^ (-1.53 - -1.28) |
|  | 1996-2008 | -1.26^*^ (-1.44 - -1.09) |  |
|  | 2008-2021 | -1.97^*^ (-2.12 - -1.82) |  |
| Chronic obstructive pulmonary disease | 1990-1997 | 0.33 (-0.25 - 0.91) | -0.79^*^ (-1.38 - -0.20) |
|  | 1997-2000 | -3.15 (-6.93 - 0.78) |  |
|  | 2000-2008 | 0.58^*^ (0.08 - 1.09) |  |
|  | 2008-2011 | -2.39 (-5.68 - 1.02) |  |
|  | 2011-2014 | 1.99 (-1.51 - 5.62) |  |
|  | 2014-2021 | -2.89^*^ (-3.39 - -2.39) |  |
| Lower respiratory infections | 1990-2006 | -2.06^*^ (-2.27 - -1.85) | -2.86^*^ (-3.17 - -2.55) |
|  | 2006-2018 | -2.56^*^ (-2.93 - -2.19) |  |
|  | 2018-2021 | -8.16^*^ (-10.84 - -5.40) |  |
| Neonatal disorders | 1990-1994 | -0.64^*^ (-0.98 - -0.30) | -1.99^*^ (-2.17 - -1.80) |
|  | 1994-2006 | -1.83^*^ (-1.90 - -1.75) |  |
|  | 2006-2016 | -2.04^*^ (-2.16 - -1.92) |  |
|  | 2016-2019 | -3.90^*^ (-5.36 - -2.42) |  |
|  | 2019-2021 | -2.43^*^ (-4.29 - -0.53) |  |
| **Low SDI** |  |  |  |
| Ischemic heart disease | 1990-1997 | 0.34^*^ (0.01 - 0.67) | -0.17 (-0.40 - 0.06) |
|  | 1997-2006 | -0.76^*^ (-1.01 - -0.50) |  |
|  | 2006-2010 | -0.05 (-1.23 - 1.14) |  |
|  | 2010-2014 | 2.78^*^ (1.57 - 4.01) |  |
|  | 2014-2021 | -1.65^*^ (-1.96 - -1.33) |  |
| Stroke | 1990-1996 | -0.19 (-0.38 - 0.01) | -1.08^*^ (-1.21 - -0.94) |
|  | 1996-2007 | -1.68^*^ (-1.76 - -1.59) |  |
|  | 2007-2011 | -1.23^*^ (-1.79 - -0.67) |  |
|  | 2011-2014 | 0.86 (-0.27 - 2.00) |  |
|  | 2014-2021 | -1.63^*^ (-1.79 - -1.47) |  |
| Chronic obstructive pulmonary disease | 1990-1995 | 1.16^*^ (0.20 - 2.12) | -0.39 (-0.82 - 0.03) |
|  | 1995-1999 | -2.40^*^ (-4.32 - -0.44) |  |
|  | 1999-2011 | -0.12 (-0.37 - 0.13) |  |
|  | 2011-2014 | 4.57^*^ (1.14 - 8.11) |  |
|  | 2014-2021 | -2.85^*^ (-3.27 - -2.43) |  |
| Lower respiratory infections | 1990-1995 | -0.51 (-1.02 - 0.00) | -2.42^*^ (-2.71 - -2.12) |
|  | 1995-2001 | -2.09^*^ (-2.58 - -1.59) |  |
|  | 2001-2011 | -2.93^*^ (-3.13 - -2.73) |  |
|  | 2011-2014 | 0.43 (-1.83 - 2.75) |  |
|  | 2014-2018 | -3.00^*^ (-4.11 - -1.86) |  |
|  | 2018-2021 | -6.46^*^ (-7.56 - -5.34) |  |
| Neonatal disorders | 1990-2002 | -1.01^*^ (-1.04 - -0.99) | -1.22^*^ (-1.27 - -1.17) |
|  | 2002-2005 | -0.56^*^ (-1.02 - -0.09) |  |
|  | 2005-2015 | -0.94^*^ (-0.99 - -0.89) |  |
|  | 2015-2021 | -2.42^*^ (-2.56 - -2.29) |  |
| **High-income** |  |  |  |
| Ischemic heart disease | 1990-1998 | -3.97^*^ (-4.24 - -3.70) | -5.48^*^ (-5.70 - -5.26) |
|  | 1998-2003 | -5.42^*^ (-6.01 - -4.82) |  |
|  | 2003-2010 | -7.43^*^ (-7.76 - -7.09) |  |
|  | 2010-2019 | -6.09^*^ (-6.34 - -5.84) |  |
|  | 2019-2021 | -1.95 (-4.71 - 0.88) |  |
| Stroke | 1990-1999 | -4.06^*^ (-4.17 - -3.95) | -5.03^*^ (-5.16 - -4.90) |
|  | 1999-2003 | -5.37^*^ (-5.84 - -4.89) |  |
|  | 2003-2010 | -7.42^*^ (-7.59 - -7.26) |  |
|  | 2010-2015 | -4.04^*^ (-4.38 - -3.70) |  |
|  | 2015-2019 | -5.63^*^ (-6.20 - -5.06) |  |
|  | 2019-2021 | -1.41^*^ (-2.71 - -0.08) |  |
| Chronic obstructive pulmonary disease | 1990-2002 | -1.86^*^ (-2.12 - -1.60) | -3.30^*^ (-3.63 - -2.97) |
|  | 2002-2019 | -4.49^*^ (-4.64 - -4.35) |  |
|  | 2019-2021 | -1.62 (-6.58 - 3.59) |  |
| Lower respiratory infections | 1990-1998 | -0.38 (-0.94 - 0.19) | -3.50^*^ (-4.18 - -2.81) |
|  | 1998-2001 | -6.99^*^ (-11.66 - -2.08) |  |
|  | 2001-2010 | -4.35^*^ (-4.88 - -3.82) |  |
|  | 2010-2015 | -0.65 (-2.25 - 0.98) |  |
|  | 2015-2019 | -3.72^*^ (-6.20 - -1.17) |  |
|  | 2019-2021 | -12.70^*^ (-17.28 - -7.88) |  |
| Neonatal disorders | 1990-2000 | -4.50^*^ (-4.62 - -4.38) | -4.36^*^ (-4.61 - -4.10) |
|  | 2000-2003 | -2.69^*^ (-4.20 - -1.17) |  |
|  | 2003-2007 | -5.20^*^ (-5.93 - -4.46) |  |
|  | 2007-2010 | -6.20^*^ (-7.64 - -4.74) |  |
|  | 2010-2019 | -3.22^*^ (-3.40 - -3.04) |  |
|  | 2019-2021 | -6.71^*^ (-8.89 - -4.48) |  |
| **Southeast Asia, East Asia, and Oceania** |  |  |  |
| Ischemic heart disease | 1990-1998 | -0.27 (-0.79 - 0.26) | -0.52^*^ (-0.82 - -0.22) |
|  | 1998-2004 | 3.31^*^ (2.24 - 4.38) |  |
|  | 2004-2013 | -0.55^*^ (-1.06 - -0.04) |  |
|  | 2013-2021 | -3.52^*^ (-4.09 - -2.96) |  |
| Stroke | 1990-1998 | -1.46^*^ (-1.89 - -1.02) | -3.13^*^ (-3.67 - -2.59) |
|  | 1998-2004 | 0.08 (-0.78 - 0.94) |  |
|  | 2004-2007 | -6.26^*^ (-9.88 - -2.49) |  |
|  | 2007-2010 | -2.98 (-6.88 - 1.09) |  |
|  | 2010-2021 | -5.22^*^ (-5.54 - -4.89) |  |
| Chronic obstructive pulmonary disease | 1990-1995 | -1.91^*^ (-2.39 - -1.43) | -5.02^*^ (-5.46 - -4.58) |
|  | 1995-2004 | -3.47^*^ (-3.70 - -3.24) |  |
|  | 2004-2007 | -9.36^*^ (-11.57 - -7.10) |  |
|  | 2007-2010 | -5.04^*^ (-7.62 - -2.37) |  |
|  | 2010-2019 | -7.34^*^ (-7.71 - -6.97) |  |
|  | 2019-2021 | -2.27 (-6.97 - 2.67) |  |
| Lower respiratory infections | 1990-1996 | -3.26^*^ (-4.00 - -2.52) | -4.76^*^ (-4.92 - -4.61) |
|  | 1996-2021 | -5.12^*^ (-5.21 - -5.03) |  |
| Neonatal disorders | 1990-1995 | -1.09^*^ (-1.70 - -0.48) | -3.80^*^ (-4.04 - -3.57) |
|  | 1995-2003 | -3.03^*^ (-3.36 - -2.69) |  |
|  | 2003-2011 | -4.74^*^ (-5.10 - -4.38) |  |
|  | 2011-2017 | -6.65^*^ (-7.27 - -6.01) |  |
|  | 2017-2021 | -2.45^*^ (-3.60 - -1.28) |  |
| **Central Europe, Eastern Europe, and Central Asia** |  |  |  |
| Ischemic heart disease | 1990-1994 | 4.13^*^ (2.15 - 6.16) | -3.00^*^ (-3.37 - -2.64) |
|  | 1994-2002 | -2.03^*^ (-2.78 - -1.28) |  |
|  | 2002-2010 | -4.15^*^ (-4.85 - -3.44) |  |
|  | 2010-2021 | -5.35^*^ (-5.72 - -4.97) |  |
| Stroke | 1990-1994 | 3.30^*^ (1.82 - 4.79) | -3.98^*^ (-4.32 - -3.65) |
|  | 1994-2002 | -2.70^*^ (-3.23 - -2.16) |  |
|  | 2002-2019 | -6.54^*^ (-6.67 - -6.40) |  |
|  | 2019-2021 | -1.10 (-5.31 - 3.29) |  |
| Chronic obstructive pulmonary disease | 1990-1994 | 0.66 (-1.02 - 2.37) | -4.92^*^ (-5.43 - -4.41) |
|  | 1994-2001 | -4.75^*^ (-5.51 - -3.97) |  |
|  | 2001-2010 | -7.94^*^ (-8.39 - -7.49) |  |
|  | 2010-2015 | -3.99^*^ (-5.32 - -2.64) |  |
|  | 2015-2019 | -6.90^*^ (-9.00 - -4.75) |  |
|  | 2019-2021 | -0.81 (-5.57 - 4.19) |  |
| Lower respiratory infections | 1990-1995 | 4.62^*^ (3.71 - 5.53) | -2.88^*^ (-3.23 - -2.52) |
|  | 1995-2000 | -2.33^*^ (-3.52 - -1.11) |  |
|  | 2000-2009 | -4.88^*^ (-5.29 - -4.47) |  |
|  | 2009-2019 | -3.18^*^ (-3.53 - -2.83) |  |
|  | 2019-2021 | -11.30^*^ (-14.80 - -7.65) |  |
| Neonatal disorders | 1990-1994 | 0.43 (-0.26 - 1.13) | -2.81^*^ (-3.00 - -2.62) |
|  | 1994-2000 | -2.82^*^ (-3.33 - -2.31) |  |
|  | 2000-2004 | -4.98^*^ (-6.07 - -3.89) |  |
|  | 2004-2021 | -3.04^*^ (-3.16 - -2.92) |  |
| **Latin America and Caribbean** |  |  |  |
| Ischemic heart disease | 1990-1995 | -2.49^*^ (-2.91 - -2.06) | -2.96^*^ (-3.25 - -2.67) |
|  | 1995-2007 | -3.63^*^ (-3.75 - -3.51) |  |
|  | 2007-2013 | -2.69^*^ (-3.11 - -2.28) |  |
|  | 2013-2016 | -1.67 (-3.55 - 0.25) |  |
|  | 2016-2019 | -4.18^*^ (-6.12 - -2.21) |  |
|  | 2019-2021 | -1.00 (-3.11 - 1.16) |  |
| Stroke | 1990-1994 | -3.15^*^ (-3.85 - -2.44) | -4.24^*^ (-4.43 - -4.05) |
|  | 1994-2005 | -4.35^*^ (-4.52 - -4.19) |  |
|  | 2005-2019 | -4.82^*^ (-4.93 - -4.70) |  |
|  | 2019-2021 | -1.67 (-4.23 - 0.96) |  |
| Chronic obstructive pulmonary disease | 1990-1998 | -1.10^*^ (-1.55 - -0.65) | -3.60^*^ (-4.03 - -3.18) |
|  | 1998-2004 | -3.61^*^ (-4.50 - -2.72) |  |
|  | 2004-2007 | -5.89^*^ (-9.72 - -1.90) |  |
|  | 2007-2021 | -4.51^*^ (-4.72 - -4.30) |  |
| Lower respiratory infections | 1990-1995 | -3.06^*^ (-3.76 - -2.35) | -3.65^*^ (-3.96 - -3.35) |
|  | 1995-2000 | -5.04^*^ (-6.01 - -4.06) |  |
|  | 2000-2010 | -2.94^*^ (-3.23 - -2.65) |  |
|  | 2010-2019 | -2.02^*^ (-2.38 - -1.66) |  |
|  | 2019-2021 | -12.13^*^ (-15.22 - -8.93) |  |
| Neonatal disorders | 1990-1994 | -3.79^*^ (-4.03 - -3.54) | -4.34^*^ (-4.46 - -4.22) |
|  | 1994-2001 | -3.43^*^ (-3.57 - -3.28) |  |
|  | 2001-2004 | -4.40^*^ (-5.22 - -3.57) |  |
|  | 2004-2011 | -5.26^*^ (-5.43 - -5.08) |  |
|  | 2011-2017 | -4.02^*^ (-4.29 - -3.75) |  |
|  | 2017-2021 | -5.28^*^ (-5.78 - -4.78) |  |
| **North Africa and Middle East** |  |  |  |
| Ischemic heart disease | 1990-2015 | -1.03^*^ (-1.09 - -0.96) | -1.22^*^ (-1.33 - -1.10) |
|  | 2015-2021 | -2.01^*^ (-2.56 - -1.45) |  |
| Stroke | 1990-1999 | -1.63^*^ (-1.98 - -1.28) | -2.07^*^ (-2.31 - -1.83) |
|  | 1999-2005 | -2.70^*^ (-3.55 - -1.85) |  |
|  | 2005-2015 | -1.53^*^ (-1.86 - -1.19) |  |
|  | 2015-2021 | -2.98^*^ (-3.60 - -2.35) |  |
| Chronic obstructive pulmonary disease | 1990-2005 | -2.31^*^ (-2.55 - -2.07) | -1.89^*^ (-2.11 - -1.67) |
|  | 2005-2015 | -0.56^*^ (-0.97 - -0.13) |  |
|  | 2015-2021 | -3.04^*^ (-3.77 - -2.31) |  |
| Lower respiratory infections | 1990-2012 | -2.62^*^ (-2.66 - -2.58) | -3.05^*^ (-3.26 - -2.83) |
|  | 2012-2016 | -1.66^*^ (-2.50 - -0.82) |  |
|  | 2016-2019 | -3.13^*^ (-4.79 - -1.45) |  |
|  | 2019-2021 | -10.09^*^ (-11.65 - -8.49) |  |
| Neonatal disorders | 1990-2002 | -1.81^*^ (-1.94 - -1.68) | -3.23^*^ (-3.33 - -3.13) |
|  | 2002-2010 | -3.03^*^ (-3.31 - -2.75) |  |
|  | 2010-2021 | -4.91^*^ (-5.08 - -4.75) |  |
| **South Asia** |  |  |  |
| Ischemic heart disease | 1990-1997 | 0.62^*^ (0.01 - 1.23) | -0.11 (-0.70 - 0.48) |
|  | 1997-2000 | -2.13 (-6.33 - 2.27) |  |
|  | 2000-2011 | 0.23 (-0.10 - 0.57) |  |
|  | 2011-2014 | 4.49^*^ (0.09 - 9.08) |  |
|  | 2014-2021 | -2.42^*^ (-3.00 - -1.84) |  |
| Stroke | 1990-1996 | 0.03 (-0.71 - 0.77) | -1.32^*^ (-1.94 - -0.70) |
|  | 1996-2000 | -2.61^*^ (-4.64 - -0.53) |  |
|  | 2000-2008 | -1.17^*^ (-1.72 - -0.62) |  |
|  | 2008-2011 | -3.23 (-7.15 - 0.86) |  |
|  | 2011-2014 | 1.58 (-2.65 - 6.00) |  |
|  | 2014-2021 | -2.30^*^ (-2.88 - -1.73) |  |
| Chronic obstructive pulmonary disease | 1990-1996 | 0.78 (-0.16 - 1.74) | -0.90^*^ (-1.55 - -0.24) |
|  | 1996-2000 | -3.22^*^ (-5.64 - -0.73) |  |
|  | 2000-2008 | 0.46 (-0.16 - 1.08) |  |
|  | 2008-2011 | -2.91 (-6.83 - 1.17) |  |
|  | 2011-2014 | 3.82 (-0.48 - 8.30) |  |
|  | 2014-2021 | -3.60^*^ (-4.18 - -3.02) |  |
| Lower respiratory infections | 1990-2017 | -2.15^*^ (-2.29 - -2.02) | -2.88^*^ (-3.23 - -2.54) |
|  | 2017-2021 | -7.67^*^ (-10.14 - -5.13) |  |
| Neonatal disorders | 1990-1993 | -0.56 (-1.36 - 0.24) | -1.84^*^ (-1.99 - -1.70) |
|  | 1993-2009 | -1.77^*^ (-1.85 - -1.70) |  |
|  | 2009-2015 | -1.16^*^ (-1.62 - -0.69) |  |
|  | 2015-2021 | -3.34^*^ (-3.76 - -2.92) |  |
| **Sub-Saharan Africa** |  |  |  |
| Ischemic heart disease | 1990-1994 | -0.00 (-0.76 - 0.76) | -0.18^*^ (-0.35 - -0.01) |
|  | 1994-2000 | 1.52^*^ (0.98 - 2.06) |  |
|  | 2000-2006 | -1.26^*^ (-1.78 - -0.73) |  |
|  | 2006-2021 | -0.46^*^ (-0.56 - -0.36) |  |
| Stroke | 1990-1998 | -0.29^*^ (-0.36 - -0.21) | -1.10^*^ (-1.18 - -1.01) |
|  | 1998-2002 | -1.03^*^ (-1.38 - -0.68) |  |
|  | 2002-2007 | -1.64^*^ (-1.87 - -1.42) |  |
|  | 2007-2015 | -1.29^*^ (-1.38 - -1.19) |  |
|  | 2015-2019 | -1.87^*^ (-2.24 - -1.49) |  |
|  | 2019-2021 | -0.78 (-1.57 - 0.01) |  |
| Chronic obstructive pulmonary disease | 1990-1997 | -0.38^*^ (-0.46 - -0.31) | -1.05^*^ (-1.11 - -1.00) |
|  | 1997-2008 | -1.30^*^ (-1.34 - -1.26) |  |
|  | 2008-2015 | -1.14^*^ (-1.23 - -1.04) |  |
|  | 2015-2019 | -1.55^*^ (-1.84 - -1.26) |  |
|  | 2019-2021 | -0.76^*^ (-1.35 - -0.17) |  |
| Lower respiratory infections | 1990-2000 | -0.66^*^ (-0.75 - -0.57) | -2.17^*^ (-2.31 - -2.03) |
|  | 2000-2010 | -2.53^*^ (-2.64 - -2.43) |  |
|  | 2010-2016 | -1.79^*^ (-2.05 - -1.53) |  |
|  | 2016-2019 | -3.74^*^ (-4.89 - -2.57) |  |
|  | 2019-2021 | -6.56^*^ (-7.69 - -5.42) |  |
| Neonatal disorders | 1990-2003 | -0.65^*^ (-0.67 - -0.63) | -1.06^*^ (-1.13 - -0.98) |
|  | 2003-2006 | -0.54^*^ (-0.92 - -0.16) |  |
|  | 2006-2016 | -0.97^*^ (-1.01 - -0.93) |  |
|  | 2016-2019 | -2.85^*^ (-3.42 - -2.27) |  |
|  | 2019-2021 | -2.19^*^ (-2.89 - -1.49) |  |

ASMR: age-standardized mortality rates; APC: annual percentage change; AAPC: average annual percentage change; CI: confidence interval; SDI: socio-demographic index; ^*^, *P* < 0.05.
