# Supplementary material for: Temporal trends of particulate matter pollution and its health burden, 1990–2021, with projections to 2036: a systematic analysis for the global burden of disease study 2021
Source: Front Public Health. 2025 Apr 16;13:1579716. doi: 10.3389/fpubh.2025.1579716 (PMC12041061; doi:10.3389/fpubh.2025.1579716)
Supplement: Supplementary file 14 [file Table_7.DOCX]

| **Table S7. Joinpoint regression analysis: trends in ASDR of top five disease across global, five SDI quintiles, and seven GBD super regions** | | | |
| --- | --- | --- | --- |
| **Disease type** | **Period** | **APC(95%CI)** | **AAPC(95%CI)** |
| **Global** |  |  |  |
| Ischemic heart disease | 1990-1994 | 0.17 (-0.91 - 1.27) | -1.31^*^ (-1.49 - -1.13) |
|  | 1994-2015 | -1.26^*^ (-1.35 - -1.17) |  |
|  | 2015-2021 | -2.47^*^ (-3.04 - -1.89) |  |
| Stroke | 1990-2004 | -1.26^*^ (-1.33 - -1.18) | -2.36^*^ (-2.61 - -2.11) |
|  | 2004-2007 | -4.23^*^ (-5.69 - -2.74) |  |
|  | 2007-2016 | -2.99^*^ (-3.17 - -2.81) |  |
|  | 2016-2019 | -4.56^*^ (-6.28 - -2.80) |  |
|  | 2019-2021 | -1.07 (-2.99 - 0.89) |  |
| Chronic obstructive pulmonary disease | 1990-1995 | -0.87^*^ (-1.41 - -0.33) | -2.67^*^ (-2.93 - -2.41) |
|  | 1995-2004 | -2.41^*^ (-2.67 - -2.15) |  |
|  | 2004-2007 | -4.34^*^ (-6.78 - -1.85) |  |
|  | 2007-2021 | -3.11^*^ (-3.25 - -2.96) |  |
| Lower respiratory infections | 1990-1998 | -2.15^*^ (-2.60 - -1.71) | -4.01^*^ (-4.24 - -3.78) |
|  | 1998-2018 | -3.85^*^ (-3.97 - -3.73) |  |
|  | 2018-2021 | -9.78^*^ (-11.70 - -7.83) |  |
| Neonatal disorders | 1990-1994 | -0.09 (-0.33 - 0.14) | -1.42^*^ (-1.53 - -1.31) |
|  | 1994-1999 | -0.95^*^ (-1.21 - -0.70) |  |
|  | 1999-2005 | -1.46^*^ (-1.65 - -1.28) |  |
|  | 2005-2014 | -1.71^*^ (-1.81 - -1.62) |  |
|  | 2014-2018 | -2.62^*^ (-3.17 - -2.06) |  |
|  | 2018-2021 | -1.40^*^ (-2.08 - -0.72) |  |
| **High SDI** |  |  |  |
| Ischemic heart disease | 1990-1993 | -3.26^*^ (-4.25 - -2.27) | -4.17^*^ (-4.40 - -3.93) |
|  | 1993-2006 | -4.52^*^ (-4.62 - -4.42) |  |
|  | 2006-2010 | -5.60^*^ (-6.37 - -4.82) |  |
|  | 2010-2016 | -3.92^*^ (-4.28 - -3.55) |  |
|  | 2016-2019 | -4.71^*^ (-6.37 - -3.01) |  |
|  | 2019-2021 | -0.20 (-2.03 - 1.68) |  |
| Stroke | 1990-2005 | -3.38^*^ (-3.44 - -3.31) | -3.68^*^ (-3.85 - -3.51) |
|  | 2005-2010 | -5.95^*^ (-6.35 - -5.56) |  |
|  | 2010-2016 | -2.78^*^ (-3.06 - -2.50) |  |
|  | 2016-2019 | -5.32^*^ (-6.57 - -4.05) |  |
|  | 2019-2021 | -0.34 (-1.80 - 1.15) |  |
| Chronic obstructive pulmonary disease | 1990-2002 | -2.41^*^ (-2.52 - -2.31) | -3.19^*^ (-3.45 - -2.92) |
|  | 2002-2005 | -3.10^*^ (-4.55 - -1.63) |  |
|  | 2005-2010 | -5.03^*^ (-5.51 - -4.55) |  |
|  | 2010-2016 | -3.02^*^ (-3.40 - -2.64) |  |
|  | 2016-2019 | -5.59^*^ (-7.39 - -3.76) |  |
|  | 2019-2021 | -0.10 (-2.12 - 1.96) |  |
| Lower respiratory infections | 1990-1998 | -2.48^*^ (-2.89 - -2.06) | -3.94^*^ (-4.52 - -3.36) |
|  | 1998-2001 | -7.25^*^ (-10.83 - -3.53) |  |
|  | 2001-2011 | -3.50^*^ (-3.84 - -3.16) |  |
|  | 2011-2016 | -1.77^*^ (-2.98 - -0.55) |  |
|  | 2016-2019 | -4.52^*^ (-8.31 - -0.58) |  |
|  | 2019-2021 | -11.15^*^ (-14.70 - -7.46) |  |
| Neonatal disorders | 1990-1999 | -4.74^*^ (-5.01 - -4.47) | -4.89^*^ (-5.14 - -4.64) |
|  | 1999-2006 | -3.52^*^ (-4.06 - -2.98) |  |
|  | 2006-2011 | -6.50^*^ (-7.33 - -5.66) |  |
|  | 2011-2017 | -3.66^*^ (-4.25 - -3.08) |  |
|  | 2017-2021 | -7.38^*^ (-8.41 - -6.34) |  |
| **High-middle SDI** |  |  |  |
| Ischemic heart disease | 1990-1994 | 3.00^*^ (1.82 - 4.20) | -2.25^*^ (-2.69 - -1.82) |
|  | 1994-1998 | -2.90^*^ (-4.66 - -1.12) |  |
|  | 1998-2002 | -0.07 (-1.68 - 1.56) |  |
|  | 2002-2015 | -3.37^*^ (-3.56 - -3.19) |  |
|  | 2015-2019 | -5.53^*^ (-7.11 - -3.93) |  |
|  | 2019-2021 | -1.53 (-5.18 - 2.27) |  |
| Stroke | 1990-1994 | -0.15 (-0.98 - 0.68) | -3.36^*^ (-3.73 - -2.98) |
|  | 1994-2004 | -1.51^*^ (-1.74 - -1.28) |  |
|  | 2004-2008 | -6.88^*^ (-8.10 - -5.64) |  |
|  | 2008-2016 | -4.52^*^ (-4.88 - -4.15) |  |
|  | 2016-2019 | -7.24^*^ (-9.93 - -4.46) |  |
|  | 2019-2021 | -1.00 (-4.05 - 2.14) |  |
| Chronic obstructive pulmonary disease | 1990-1994 | -0.96^*^ (-1.59 - -0.34) | -4.74^*^ (-5.10 - -4.37) |
|  | 1994-2001 | -3.09^*^ (-3.44 - -2.74) |  |
|  | 2001-2005 | -5.46^*^ (-6.63 - -4.27) |  |
|  | 2005-2008 | -10.28^*^ (-12.73 - -7.75) |  |
|  | 2008-2019 | -5.81^*^ (-6.04 - -5.58) |  |
|  | 2019-2021 | -1.87 (-5.31 - 1.69) |  |
| Lower respiratory infections | 1990-1995 | -3.34^*^ (-4.30 - -2.36) | -6.39^*^ (-6.76 - -6.02) |
|  | 1995-2000 | -6.63^*^ (-7.95 - -5.29) |  |
|  | 2000-2008 | -8.94^*^ (-9.50 - -8.38) |  |
|  | 2008-2018 | -5.14^*^ (-5.54 - -4.73) |  |
|  | 2018-2021 | -8.25^*^ (-10.46 - -5.99) |  |
| Neonatal disorders | 1990-1995 | -1.72^*^ (-2.50 - -0.93) | -5.68^*^ (-5.98 - -5.37) |
|  | 1995-2001 | -4.45^*^ (-5.25 - -3.63) |  |
|  | 2001-2010 | -6.83^*^ (-7.19 - -6.48) |  |
|  | 2010-2014 | -5.24^*^ (-6.81 - -3.63) |  |
|  | 2014-2021 | -8.21^*^ (-8.71 - -7.71) |  |
| **Middle SDI** |  |  |  |
| Ischemic heart disease | 1990-2004 | -0.33^*^ (-0.50 - -0.15) | -1.01^*^ (-1.19 - -0.84) |
|  | 2004-2014 | -0.71^*^ (-1.05 - -0.37) |  |
|  | 2014-2021 | -2.81^*^ (-3.34 - -2.27) |  |
| Stroke | 1990-2004 | -1.59^*^ (-1.77 - -1.41) | -3.08^*^ (-3.29 - -2.88) |
|  | 2004-2013 | -3.86^*^ (-4.31 - -3.41) |  |
|  | 2013-2021 | -4.77^*^ (-5.34 - -4.20) |  |
| Chronic obstructive pulmonary disease | 1990-1995 | -1.87^*^ (-2.59 - -1.15) | -4.43^*^ (-4.93 - -3.92) |
|  | 1995-2004 | -3.49^*^ (-3.82 - -3.15) |  |
|  | 2004-2007 | -7.19^*^ (-10.46 - -3.79) |  |
|  | 2007-2010 | -3.77 (-7.60 - 0.21) |  |
|  | 2010-2021 | -5.74^*^ (-6.13 - -5.34) |  |
| Lower respiratory infections | 1990-1995 | -4.40^*^ (-5.35 - -3.43) | -5.62^*^ (-5.92 - -5.32) |
|  | 1995-2006 | -6.03^*^ (-6.37 - -5.70) |  |
|  | 2006-2018 | -4.71^*^ (-5.01 - -4.40) |  |
|  | 2018-2021 | -9.66^*^ (-11.84 - -7.43) |  |
| Neonatal disorders | 1990-2000 | -1.95^*^ (-2.11 - -1.78) | -3.26^*^ (-3.38 - -3.15) |
|  | 2000-2014 | -3.24^*^ (-3.36 - -3.12) |  |
|  | 2014-2021 | -5.17^*^ (-5.58 - -4.75) |  |
| **Low-middle SDI** |  |  |  |
| Ischemic heart disease | 1990-1997 | 0.02 (-0.35 - 0.39) | -0.44^*^ (-0.60 - -0.28) |
|  | 1997-2005 | -0.66^*^ (-1.01 - -0.31) |  |
|  | 2005-2014 | 0.41^*^ (0.12 - 0.70) |  |
|  | 2014-2021 | -1.71^*^ (-2.07 - -1.35) |  |
| Stroke | 1990-1996 | -0.51^*^ (-0.88 - -0.14) | -1.49^*^ (-1.58 - -1.39) |
|  | 1996-2008 | -1.32^*^ (-1.47 - -1.18) |  |
|  | 2008-2021 | -2.08^*^ (-2.20 - -1.96) |  |
| Chronic obstructive pulmonary disease | 1990-1997 | 0.10 (-0.36 - 0.57) | -0.95^*^ (-1.30 - -0.59) |
|  | 1997-2000 | -2.28 (-5.45 - 1.00) |  |
|  | 2000-2008 | -0.19 (-0.60 - 0.21) |  |
|  | 2008-2015 | -1.05^*^ (-1.53 - -0.56) |  |
|  | 2015-2021 | -2.38^*^ (-2.91 - -1.85) |  |
| Lower respiratory infections | 1990-2003 | -2.85^*^ (-3.10 - -2.61) | -4.13^*^ (-4.40 - -3.87) |
|  | 2003-2018 | -3.77^*^ (-3.99 - -3.55) |  |
|  | 2018-2021 | -11.20^*^ (-13.47 - -8.88) |  |
| Neonatal disorders | 1990-1994 | -0.64^*^ (-0.98 - -0.30) | -1.99^*^ (-2.17 - -1.80) |
|  | 1994-2006 | -1.83^*^ (-1.90 - -1.75) |  |
|  | 2006-2016 | -2.04^*^ (-2.16 - -1.92) |  |
|  | 2016-2019 | -3.90^*^ (-5.36 - -2.42) |  |
|  | 2019-2021 | -2.43^*^ (-4.29 - -0.53) |  |
| **Low SDI** |  |  |  |
| Ischemic heart disease | 1990-1998 | 0.30^*^ (0.06 - 0.54) | -0.34^*^ (-0.55 - -0.13) |
|  | 1998-2006 | -1.12^*^ (-1.40 - -0.84) |  |
|  | 2006-2010 | -0.20 (-1.25 - 0.86) |  |
|  | 2010-2014 | 1.50^*^ (0.44 - 2.57) |  |
|  | 2014-2021 | -1.28^*^ (-1.56 - -1.00) |  |
| Stroke | 1990-1997 | -0.37^*^ (-0.51 - -0.23) | -1.20^*^ (-1.26 - -1.13) |
|  | 1997-2009 | -1.86^*^ (-1.92 - -1.79) |  |
|  | 2009-2015 | -0.46^*^ (-0.69 - -0.24) |  |
|  | 2015-2021 | -1.56^*^ (-1.74 - -1.38) |  |
| Chronic obstructive pulmonary disease | 1990-1995 | 0.70^*^ (0.04 - 1.36) | -0.52^*^ (-0.80 - -0.24) |
|  | 1995-2000 | -1.34^*^ (-2.19 - -0.49) |  |
|  | 2000-2011 | -0.48^*^ (-0.68 - -0.28) |  |
|  | 2011-2014 | 1.95 (-0.45 - 4.41) |  |
|  | 2014-2021 | -1.91^*^ (-2.21 - -1.60) |  |
| Lower respiratory infections | 1990-1995 | -1.48^*^ (-1.63 - -1.33) | -3.60^*^ (-3.67 - -3.52) |
|  | 1995-2001 | -2.74^*^ (-2.88 - -2.60) |  |
|  | 2001-2011 | -4.00^*^ (-4.06 - -3.94) |  |
|  | 2011-2015 | -1.68^*^ (-2.00 - -1.35) |  |
|  | 2015-2019 | -5.24^*^ (-5.56 - -4.92) |  |
|  | 2019-2021 | -9.69^*^ (-10.30 - -9.08) |  |
| Neonatal disorders | 1990-2002 | -1.01^*^ (-1.04 - -0.99) | -1.22^*^ (-1.27 - -1.17) |
|  | 2002-2005 | -0.56^*^ (-1.02 - -0.09) |  |
|  | 2005-2015 | -0.94^*^ (-0.99 - -0.89) |  |
|  | 2015-2021 | -2.42^*^ (-2.56 - -2.29) |  |
| **High-income** |  |  |  |
| Ischemic heart disease | 1990-1998 | -4.13^*^ (-4.42 - -3.85) | -5.43^*^ (-5.66 - -5.20) |
|  | 1998-2003 | -5.36^*^ (-5.97 - -4.73) |  |
|  | 2003-2010 | -7.35^*^ (-7.69 - -7.00) |  |
|  | 2010-2019 | -5.85^*^ (-6.11 - -5.58) |  |
|  | 2019-2021 | -2.05 (-4.96 - 0.95) |  |
| Stroke | 1990-1999 | -3.85^*^ (-3.95 - -3.76) | -4.70^*^ (-4.81 - -4.58) |
|  | 1999-2003 | -5.00^*^ (-5.41 - -4.58) |  |
|  | 2003-2010 | -7.06^*^ (-7.20 - -6.92) |  |
|  | 2010-2015 | -3.68^*^ (-3.96 - -3.40) |  |
|  | 2015-2019 | -5.05^*^ (-5.53 - -4.57) |  |
|  | 2019-2021 | -1.29^*^ (-2.43 - -0.13) |  |
| Chronic obstructive pulmonary disease | 1990-2002 | -1.81^*^ (-2.03 - -1.60) | -3.16^*^ (-3.43 - -2.88) |
|  | 2002-2019 | -4.28^*^ (-4.40 - -4.16) |  |
|  | 2019-2021 | -1.54 (-5.68 - 2.79) |  |
| Lower respiratory infections | 1990-1998 | -1.44^*^ (-1.95 - -0.93) | -4.01^*^ (-4.71 - -3.30) |
|  | 1998-2001 | -7.56^*^ (-11.84 - -3.08) |  |
|  | 2001-2011 | -4.25^*^ (-4.66 - -3.83) |  |
|  | 2011-2016 | -1.14 (-2.63 - 0.38) |  |
|  | 2016-2019 | -4.57 (-9.14 - 0.22) |  |
|  | 2019-2021 | -13.29^*^ (-17.53 - -8.83) |  |
| Neonatal disorders | 1990-2000 | -4.50^*^ (-4.62 - -4.38) | -4.36^*^ (-4.61 - -4.10) |
|  | 2000-2003 | -2.69^*^ (-4.20 - -1.17) |  |
|  | 2003-2007 | -5.20^*^ (-5.93 - -4.45) |  |
|  | 2007-2010 | -6.20^*^ (-7.64 - -4.73) |  |
|  | 2010-2019 | -3.22^*^ (-3.40 - -3.04) |  |
|  | 2019-2021 | -6.70^*^ (-8.88 - -4.47) |  |
| **Southeast Asia, East Asia, and Oceania** |  |  |  |
| Ischemic heart disease | 1990-1998 | -0.55^*^ (-1.00 - -0.10) | -0.87^*^ (-1.13 - -0.61) |
|  | 1998-2004 | 2.28^*^ (1.37 - 3.20) |  |
|  | 2004-2013 | -0.89^*^ (-1.33 - -0.45) |  |
|  | 2013-2021 | -3.47^*^ (-3.96 - -2.99) |  |
| Stroke | 1990-1998 | -1.72^*^ (-1.89 - -1.55) | -3.10^*^ (-3.37 - -2.83) |
|  | 1998-2004 | -0.47^*^ (-0.81 - -0.14) |  |
|  | 2004-2007 | -5.32^*^ (-6.79 - -3.83) |  |
|  | 2007-2016 | -4.29^*^ (-4.47 - -4.10) |  |
|  | 2016-2019 | -7.29^*^ (-9.16 - -5.37) |  |
|  | 2019-2021 | -1.21 (-3.45 - 1.08) |  |
| Chronic obstructive pulmonary disease | 1990-1994 | -1.87^*^ (-2.48 - -1.26) | -5.00^*^ (-5.36 - -4.63) |
|  | 1994-2004 | -3.68^*^ (-3.85 - -3.51) |  |
|  | 2004-2007 | -8.79^*^ (-10.78 - -6.75) |  |
|  | 2007-2011 | -5.49^*^ (-6.68 - -4.29) |  |
|  | 2011-2019 | -7.08^*^ (-7.50 - -6.67) |  |
|  | 2019-2021 | -2.42 (-6.57 - 1.91) |  |
| Lower respiratory infections | 1990-1995 | -4.11^*^ (-4.83 - -3.38) | -6.30^*^ (-6.57 - -6.04) |
|  | 1995-2000 | -6.17^*^ (-7.17 - -5.17) |  |
|  | 2000-2007 | -7.80^*^ (-8.34 - -7.26) |  |
|  | 2007-2015 | -6.80^*^ (-7.24 - -6.36) |  |
|  | 2015-2021 | -5.78^*^ (-6.36 - -5.20) |  |
| Neonatal disorders | 1990-1995 | -1.09^*^ (-1.70 - -0.48) | -3.80^*^ (-4.04 - -3.57) |
|  | 1995-2003 | -3.03^*^ (-3.36 - -2.69) |  |
|  | 2003-2011 | -4.74^*^ (-5.10 - -4.38) |  |
|  | 2011-2017 | -6.64^*^ (-7.27 - -6.01) |  |
|  | 2017-2021 | -2.45^*^ (-3.60 - -1.28) |  |
| **Central Europe, Eastern Europe, and Central Asia** |  |  |  |
| Ischemic heart disease | 1990-1994 | 5.81^*^ (4.47 - 7.16) | -2.85^*^ (-3.29 - -2.40) |
|  | 1994-1998 | -3.49^*^ (-5.39 - -1.56) |  |
|  | 1998-2002 | -0.92 (-2.61 - 0.81) |  |
|  | 2002-2010 | -4.42^*^ (-4.89 - -3.95) |  |
|  | 2010-2019 | -5.80^*^ (-6.18 - -5.43) |  |
|  | 2019-2021 | -2.09 (-6.16 - 2.17) |  |
| Stroke | 1990-1994 | 3.94^*^ (2.40 - 5.50) | -3.78^*^ (-4.12 - -3.43) |
|  | 1994-2002 | -2.65^*^ (-3.20 - -2.10) |  |
|  | 2002-2019 | -6.33^*^ (-6.47 - -6.19) |  |
|  | 2019-2021 | -1.06 (-5.29 - 3.36) |  |
| Chronic obstructive pulmonary disease | 1990-1994 | 1.59 (-0.07 - 3.29) | -4.34^*^ (-4.84 - -3.85) |
|  | 1994-2001 | -4.26^*^ (-5.02 - -3.49) |  |
|  | 2001-2010 | -7.19^*^ (-7.64 - -6.75) |  |
|  | 2010-2015 | -3.92^*^ (-5.21 - -2.60) |  |
|  | 2015-2019 | -6.13^*^ (-8.15 - -4.08) |  |
|  | 2019-2021 | -0.51 (-5.16 - 4.38) |  |
| Lower respiratory infections | 1990-1995 | 4.08^*^ (3.45 - 4.70) | -3.72^*^ (-3.96 - -3.47) |
|  | 1995-2000 | -3.29^*^ (-4.11 - -2.46) |  |
|  | 2000-2008 | -6.34^*^ (-6.68 - -6.00) |  |
|  | 2008-2019 | -3.79^*^ (-4.00 - -3.59) |  |
|  | 2019-2021 | -12.08^*^ (-14.47 - -9.63) |  |
| Neonatal disorders | 1990-1994 | 0.43 (-0.26 - 1.13) | -2.81^*^ (-3.00 - -2.62) |
|  | 1994-2000 | -2.82^*^ (-3.33 - -2.31) |  |
|  | 2000-2004 | -4.98^*^ (-6.07 - -3.89) |  |
|  | 2004-2021 | -3.04^*^ (-3.16 - -2.92) |  |
| **Latin America and Caribbean** |  |  |  |
| Ischemic heart disease | 1990-1995 | -2.43^*^ (-2.78 - -2.09) | -2.88^*^ (-3.12 - -2.65) |
|  | 1995-2007 | -3.49^*^ (-3.59 - -3.39) |  |
|  | 2007-2013 | -2.90^*^ (-3.22 - -2.57) |  |
|  | 2013-2016 | -1.77^*^ (-3.28 - -0.24) |  |
|  | 2016-2019 | -3.93^*^ (-5.48 - -2.37) |  |
|  | 2019-2021 | -0.33 (-2.06 - 1.43) |  |
| Stroke | 1990-1994 | -3.05^*^ (-3.66 - -2.44) | -4.14^*^ (-4.33 - -3.96) |
|  | 1994-2005 | -4.34^*^ (-4.48 - -4.20) |  |
|  | 2005-2014 | -4.96^*^ (-5.16 - -4.76) |  |
|  | 2014-2019 | -4.23^*^ (-4.85 - -3.62) |  |
|  | 2019-2021 | -1.31 (-3.49 - 0.92) |  |
| Chronic obstructive pulmonary disease | 1990-1998 | -1.03^*^ (-1.38 - -0.69) | -3.35^*^ (-3.74 - -2.96) |
|  | 1998-2004 | -3.50^*^ (-4.17 - -2.83) |  |
|  | 2004-2007 | -5.52^*^ (-8.48 - -2.46) |  |
|  | 2007-2019 | -4.57^*^ (-4.79 - -4.35) |  |
|  | 2019-2021 | -1.37 (-5.01 - 2.40) |  |
| Lower respiratory infections | 1990-2000 | -5.22^*^ (-5.45 - -4.98) | -4.99^*^ (-5.24 - -4.74) |
|  | 2000-2011 | -4.29^*^ (-4.53 - -4.05) |  |
|  | 2011-2019 | -3.34^*^ (-3.76 - -2.92) |  |
|  | 2019-2021 | -13.75^*^ (-16.69 - -10.70) |  |
| Neonatal disorders | 1990-1994 | -3.79^*^ (-4.03 - -3.54) | -4.34^*^ (-4.46 - -4.22) |
|  | 1994-2001 | -3.42^*^ (-3.57 - -3.28) |  |
|  | 2001-2004 | -4.40^*^ (-5.22 - -3.57) |  |
|  | 2004-2011 | -5.25^*^ (-5.43 - -5.08) |  |
|  | 2011-2017 | -4.02^*^ (-4.29 - -3.75) |  |
|  | 2017-2021 | -5.28^*^ (-5.78 - -4.78) |  |
| **North Africa and Middle East** |  |  |  |
| Ischemic heart disease | 1990-1998 | -0.81^*^ (-1.17 - -0.45) | -1.31^*^ (-1.56 - -1.05) |
|  | 1998-2006 | -1.53^*^ (-1.97 - -1.09) |  |
|  | 2006-2010 | -0.23 (-1.90 - 1.46) |  |
|  | 2010-2021 | -1.89^*^ (-2.11 - -1.67) |  |
| Stroke | 1990-1998 | -1.50^*^ (-1.92 - -1.08) | -2.11^*^ (-2.34 - -1.88) |
|  | 1998-2005 | -2.64^*^ (-3.27 - -1.99) |  |
|  | 2005-2014 | -1.59^*^ (-2.00 - -1.19) |  |
|  | 2014-2021 | -2.95^*^ (-3.45 - -2.44) |  |
| Chronic obstructive pulmonary disease | 1990-2006 | -1.99^*^ (-2.19 - -1.78) | -1.71^*^ (-1.95 - -1.46) |
|  | 2006-2012 | -0.13 (-1.19 - 0.94) |  |
|  | 2012-2021 | -2.25^*^ (-2.65 - -1.83) |  |
| Lower respiratory infections | 1990-1992 | -4.67^*^ (-6.08 - -3.25) | -4.71^*^ (-4.87 - -4.55) |
|  | 1992-1998 | -3.09^*^ (-3.41 - -2.77) |  |
|  | 1998-2005 | -3.74^*^ (-3.98 - -3.50) |  |
|  | 2005-2012 | -4.76^*^ (-4.99 - -4.53) |  |
|  | 2012-2019 | -4.25^*^ (-4.49 - -4.01) |  |
|  | 2019-2021 | -13.91^*^ (-15.20 - -12.60) |  |
| Neonatal disorders | 1990-2002 | -1.81^*^ (-1.94 - -1.67) | -3.23^*^ (-3.33 - -3.13) |
|  | 2002-2010 | -3.02^*^ (-3.30 - -2.75) |  |
|  | 2010-2021 | -4.91^*^ (-5.08 - -4.75) |  |
| **South Asia** |  |  |  |
| Ischemic heart disease | 1990-1996 | 0.26 (-0.50 - 1.02) | -0.28 (-0.57 - 0.02) |
|  | 1996-2009 | -0.58^*^ (-0.83 - -0.32) |  |
|  | 2009-2014 | 2.14^*^ (0.78 - 3.51) |  |
|  | 2014-2021 | -1.87^*^ (-2.45 - -1.28) |  |
| Stroke | 1990-1996 | -0.53 (-1.20 - 0.14) | -1.46^*^ (-1.73 - -1.20) |
|  | 1996-2011 | -1.72^*^ (-1.89 - -1.54) |  |
|  | 2011-2017 | -0.76 (-1.63 - 0.12) |  |
|  | 2017-2021 | -2.93^*^ (-4.18 - -1.66) |  |
| Chronic obstructive pulmonary disease | 1990-1997 | 0.09 (-0.64 - 0.82) | -1.05^*^ (-1.56 - -0.54) |
|  | 1997-2000 | -2.56 (-7.39 - 2.52) |  |
|  | 2000-2015 | -0.57^*^ (-0.77 - -0.36) |  |
|  | 2015-2021 | -2.80^*^ (-3.53 - -2.07) |  |
| Lower respiratory infections | 1990-2017 | -3.10^*^ (-3.23 - -2.98) | -4.11^*^ (-4.41 - -3.81) |
|  | 2017-2021 | -10.65^*^ (-12.76 - -8.49) |  |
| Neonatal disorders | 1990-1993 | -0.56 (-1.36 - 0.24) | -1.84^*^ (-1.99 - -1.70) |
|  | 1993-2009 | -1.77^*^ (-1.85 - -1.70) |  |
|  | 2009-2015 | -1.16^*^ (-1.62 - -0.69) |  |
|  | 2015-2021 | -3.34^*^ (-3.76 - -2.92) |  |
| **Sub-Saharan Africa** |  |  |  |
| Ischemic heart disease | 1990-1994 | 0.02 (-0.70 - 0.74) | -0.29^*^ (-0.46 - -0.13) |
|  | 1994-2000 | 1.35^*^ (0.83 - 1.87) |  |
|  | 2000-2006 | -1.40^*^ (-1.91 - -0.89) |  |
|  | 2006-2021 | -0.58^*^ (-0.68 - -0.49) |  |
| Stroke | 1990-1998 | -0.40^*^ (-0.46 - -0.35) | -1.22^*^ (-1.28 - -1.15) |
|  | 1998-2002 | -1.29^*^ (-1.54 - -1.04) |  |
|  | 2002-2008 | -1.77^*^ (-1.89 - -1.65) |  |
|  | 2008-2015 | -1.30^*^ (-1.39 - -1.21) |  |
|  | 2015-2019 | -1.99^*^ (-2.26 - -1.71) |  |
|  | 2019-2021 | -0.78^*^ (-1.37 - -0.19) |  |
| Chronic obstructive pulmonary disease | 1990-1997 | -0.40^*^ (-0.47 - -0.34) | -0.92^*^ (-0.96 - -0.87) |
|  | 1997-2009 | -1.14^*^ (-1.17 - -1.10) |  |
|  | 2009-2015 | -0.96^*^ (-1.07 - -0.85) |  |
|  | 2015-2019 | -1.37^*^ (-1.61 - -1.13) |  |
|  | 2019-2021 | -0.32 (-0.82 - 0.18) |  |
| Lower respiratory infections | 1990-1994 | -1.10^*^ (-1.35 - -0.86) | -3.28^*^ (-3.38 - -3.18) |
|  | 1994-2001 | -1.95^*^ (-2.08 - -1.82) |  |
|  | 2001-2010 | -3.79^*^ (-3.87 - -3.71) |  |
|  | 2010-2016 | -2.44^*^ (-2.61 - -2.27) |  |
|  | 2016-2019 | -5.71^*^ (-6.46 - -4.96) |  |
|  | 2019-2021 | -8.61^*^ (-9.35 - -7.87) |  |
| Neonatal disorders | 1990-2003 | -0.65^*^ (-0.67 - -0.63) | -1.06^*^ (-1.13 - -0.98) |
|  | 2003-2006 | -0.54^*^ (-0.92 - -0.16) |  |
|  | 2006-2016 | -0.97^*^ (-1.01 - -0.93) |  |
|  | 2016-2019 | -2.85^*^ (-3.42 - -2.27) |  |
|  | 2019-2021 | -2.19^*^ (-2.89 - -1.49) |  |

ASDR, age-standardized DALY rates; APC: annual percentage change; AAPC: average annual percentage change; CI: confidence interval; SDI: socio-demographic index; ^*^, *P* < 0.05.
